# PD-L1 blockade TAM-dependently potentiates mild photothermal therapy against triple-negative breast cancer

**DOI:** 10.1186/s12951-023-02240-3

**Published:** 2023-12-11

**Authors:** Chao Wang, Yong-Hong Xu, Hua-Zhen Xu, Ke Li, Quan Zhang, Lin Shi, Li Zhao, Xiao Chen

**Affiliations:** 1 Department of Pharmacology, School of Basic Medical Sciences, Wuhan University, Donghu Avenue No. 185, Wuhan, 430072 China; 2 Department of Ophthalmology, Institute of Ophthalmological Research, Renmin Hospital of Wuhan University, Wuhan, 430060 China; 3 Center for Lab Teaching, School of Basic Medical Sciences, Wuhan University, Donghu Avenue No. 185, Wuhan, 430072 China; 4 Department of Anatomy and Embryology, School of Basic Medical Sciences, Wuhan University, Donghu Avenue No. 185, Wuhan, 430072 China; 5 Grand Pharma (China) Co., Ltd, Hubei, China; 6 State Key Laboratory of Radiation Medicine and Protection, School of Radiation Medicine and Protection, School for Radiological and Interdisciplinary Sciences (RAD-X), Collaborative Innovation Center of Radiation Medicine of Jiangsu Higher Education Institutions, Soochow University, Suzhou, 215123 Jiangsu China; 7 Hubei Provincial Key Laboratory of Developmentally Originated Disease, Wuhan, 430072 China

**Keywords:** Mild photothermia, Immune checkpoint inhibition, PD-L1, Triple-negative breast cancer, Tumor-associated macrophages

## Abstract

The present work was an endeavor to shed light on how mild photothermia possibly synergizes with immune checkpoint inhibition for tumor therapy. We established mild photothermal heating protocols to generate temperatures of 43 °C and 45 °C in both in vitro and in vivo mouse 4T1 triple-negative breast cancer (TNBC) models using polyglycerol-coated carbon nanohorns (CNH-PG) and 808 nm laser irradiation. Next, we found that 1) CNH-PG-mediated mild photothermia (CNH-PG-mPT) significantly increased expression of the immune checkpoint PD-L1 and type-1 macrophage (M1) markers in the TNBC tumors; 2) CNH-PG-mPT had a lower level of anti-tumor efficacy which was markedly potentiated by BMS-1, a PD-L1 blocker. These observations prompted us to explore the synergetic mechanisms of CNH-PG-mPT and BMS-1 in the context of tumor cell-macrophage interactions mediated by PD-L1 since tumor-associated macrophages (TAMs) are a major source of PD-L1 expression in tumors. In vitro, the study then identified two dimensions where BMS-1 potentiated CNH-PG-mPT. First, CNH-PG-mPT induced PD-L1 upregulation in the tumor cells and showed a low level of cytotoxicity which was potentiated by BMS-1. Second, CNH-PG-mPT skewed TAMs towards an M1-like anti-tumor phenotype with upregulated PD-L1, and BMS-1 bolstered the M1-like phenotype. The synergistic effects of BMS-1 and CNH-PG-mPT both on the tumor cells and TAMs were more pronounced when the two cell populations were in co-culture. Further in vivo study confirmed PD-L1 upregulation both in tumor cells and TAMs in the TNBC tumors following treatment of CNH-PG-mPT. Significantly, TAMs depletion largely abolished the anti-TNBC efficacy of CNH-PG-mPT alone and in synergy with BMS-1. Collectively, our findings reveal PD-L1 upregulation to be a key response of TNBC to mild photothermal stress, which plays a pro-survival role in the tumor cells while also acting as a brake on the M1-like activation of the TAMs. Blockade of mPT‑induced PD‑L1 achieves synergistic anti-TNBC efficacy by taking the intrinsic survival edge off the tumor cells on one hand and taking the brakes off the M1-like TAMs on the other. Our findings reveal a novel way (i.e. mild thermia plus PD-L1 blockade) to modulate the TAMs-tumor cell interaction to instigate a mutiny of the TAMs against their host tumor cells.

**Graphical Abstract:**

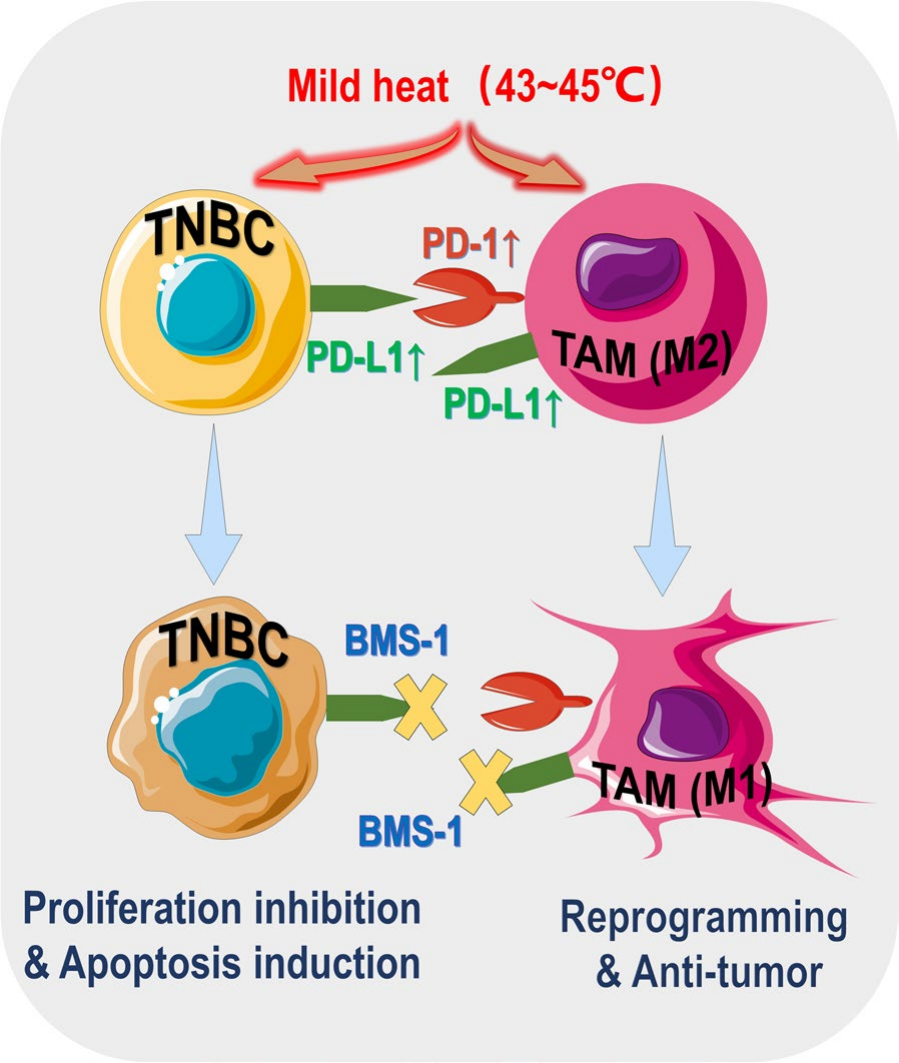

**Supplementary Information:**

The online version contains supplementary material available at 10.1186/s12951-023-02240-3.

## Introduction

In cancer, tumor cells often express programmed death-ligand 1 (PD-L1) to bind to their receptor the programmed cell death-1 (PD-1) in the tumor-infiltrating lymphocytes (TILs) to suppress their antitumor actions [1]. Therapies that block PD-L1/PD-1 thus promoting TIL-mediated anti-tumor immune responses have demonstrated remarkable therapeutic efficacy in the clinic against a range of malignant tumors [2–4]. Nonetheless, therapies blocking PD-L1/PD-1 do not work well in those immunologically “cold” tumors that are characterized by low PD-L1/PD-1 expression and/or poor lymphocyte infiltration [5–7]. Recently, tumor-associated macrophages (TAMs) have been identified as a significant source of PD-L1 and PD-1 expression in the tumor microenvironment (TME) [8, 9]. TAMs are the most abundant immune cells in the TME, making up as high as 40% of the tumor mass in certain cancers. TAMs are actively recruited into the TME and have intimate interactions with tumor cells and other tumor stromal cells. The current consensus is that TAMs mostly present an immunosuppressive phenotype, play a multitude of housekeeper functions, and suppress host anti-tumor immune responses, thereby coordinating the creation of a relatively stable environment that promotes tumor survival and growth [10–13]. Given TAMs’ servility to their host tumor cells, it would not come as a surprise if PD-L1 and PD-1 were instrumentalized by tumor cells to domesticize their TAMs. Indeed, TAMs have been identified as a major source of both PD-1 and PD-L1 expression in the TME. In colon cancer, T cell lymphoma, gastric cancer, and osteosarcoma [8, 14–16], TAMs substantially express PD-1 that sustains a pro-tumor, immunosuppressive phenotype of TAMs. PD-1 suppresses tumor cell phagocytosis by TAMs and increases secretion of IL-10 [8, 14, 17]. Surface PD-1 also acts as extrinsic handles used by tumor cells to hold back TAMs stimulation through ligation with PD-L1 [18]. On the other hand, PD-L1 is found predominantly expressed in TAMs rather than the tumor cells in non-small cell lung cancer (NSCLC), hepatocellular carcinoma, breast cancer, cholangiocarcinoma, and B-cell lymphoma [9, 19–23]. PD-L1 in TAMs, while exercising immunosuppression on other immune cells via ligation with PD-1 [24], delivers an intrinsic signal that skews TAMs towards an immunosuppressive phenotype that can be reversed by PD-L1 blockade, eliciting TAMs-mediated tumor cytotoxicity [25, 26]. Back to the side of tumor cells, PD-L1 in tumor cells, apart from ligating PD-1 of other cells, is also an intrinsic pro-survival and pro-proliferative signal that is upregulated in response to injury, for instance, sustained from radiotherapy or chemotherapy [27, 28]. In light of the above evidence, it is reasonable to envision that blockade of PD-L1 in distressed tumors may give rise to several antitumor events, i.e. 1) impaired survival and proliferative potential of the tumor cells, 2) crippled control of the TAMs by the tumor cells, and 3) reactivation of TAMs towards an immunostimulatory phenotype. These events are supposed to potentiate a distressing therapy e.g. chemotherapy or radiotherapy. There are increasing reports of therapies wherein blockade of PD-1 or PD-L1 exhibits anti-tumor efficacy dependent upon or involving TAMs repolarization [15, 16, 25, 26, 29–31]. However, the contribution of the above-proposed events to the synergetic anti-tumor efficacy brought about by PD-L1 blockade has yet to be fully confirmed and elucidated. 

Thermotherapy is a tumor treatment modality that applies high temperature to the tumor site to obtain anti-tumor efficacy. The operative temperature, heating method, and heating duration are the three key elements of thermotherapy. Different operative temperatures have been demonstrated to have distinct effects on a tumor. Temperatures above 55 °C usually kill cancer cells directly, resulting in tumor shrinkage (thermal ablation). Temperatures in the range of 39–45 °C, deemed as mild hyperthermia, are mostly applied as an auxiliary treatment to boost other therapies rather than a stand-alone therapy [32–35]. Frequently adopted heating methods include irradiation of near-infrared light (photothermal), ultrasound (sonothermal), microwave, and a magnetic field [32, 36, 37]_,_ and heating duration often falls in the 10–30 min range [32–37]. Among the subgenres of tumor thermotherapy, photothermal therapy (PTT), particularly tumor photothermal ablation, has attracted the most attention, with great promise of clinical translation. As photothermal ablation requires higher energy input and operation requirements and runs high risks of collateral damage or even destruction of normal tissues particularly adjacent to the tumor, there is growing interest in mild photothermal therapy (mPTT) of the tumors. As mentioned earlier, mPTT hardly causes direct tumor cell killing, however, there is mounting evidence that mPTT can fundamentally disturb tumor homeostasis and modulate the TME, particularly the immune compartment of the TME, into a phenotype that facilitates or enhances the efficacy of other treatment modalities, e.g. chemotherapy, radiation therapy, gene therapy, photodynamic therapy, and immune therapy [32–36, 38–40]. Of note, while there have been reports of mPTT reprogramming the immunologically “cold” TME into a “hot” one thereby sensitizing tumors to immune checkpoint inhibition, the involvement and contribution of TAMs are not clear [32, 33, 35, 36]. There are also reports that a mild elevation in tumor temperature can upregulate PD-L1 on tumor cells both to exert self-protection and immunosuppression [32, 33, 41]. Yet, the significance of these observations concerning TAMs and their interactions with the tumor cells is not clear, either.

The present work endeavored to fill the above-mentioned knowledge gaps in our understanding of mPTT and tumor immunopathology, with a special focus pinned on how mPTT would affect the cross-talk of tumor cells and TAMs mediated by PD-L1/PD-1. We first established mild photothermal heating protocols to generate temperatures of 43 °C and 45 °C in both in vitro and in vivo mouse 4T1 triple-negative breast cancer (TNBC) models using carbon nanohorns coated with polyglycerol (CNH-PG) and 808 nm laser irradiation. Next, we demonstrated the synergistic anti-TNBC efficacy of CNH-PG-mediated mild photothermia (CNH-PG-mPT) and BMS-1, a blocker of the immune checkpoint PD-L1. Mechanistic studies, both in vitro and in vivo, were then conducted to dissect the synergy, wherein the expression and functions of PD-L1 both in the tumor cells and TAMs were examined with particular regard to TAMs’ phenotype. The significance of reprogrammed TAMs in the synergy of CNH-PG-mPTT and BMS-1 was finally demonstrated in in vivo experiments. In-depth discussions were made on the novelty and significance of the obtained findings.

## Materials and methods

### CNH-PG

The synthesis and characterization of CNH-PG were described in detail in a previously published paper [42]. CNH-PG was synthesized using oxidized carbon nanohorns (CNHox, 2–5 nm in diameter and 30–50 nm in length [43]) as the starting material. It was characterized by transmission electron microscopy (TEM) and UV–vis spectroscopy. CNH-PG stock solution in water was kept at 4 °C and was sonicated in a water bath for 3 min before being diluted with culture medium or PBS into working concentrations.

### Cell models

The 4T1 mouse mammary carcinoma cell line was purchased from the Cell Bank of Shanghai Institutes for Biological Sciences (Shanghai, China). Mouse bone marrow-derived macrophages (BMDMs) were prepared according to previously published protocols [44], and type-2 activation of BMDM (BMDM2) was achieved by incubating the BMDMs with 20 ng/mL of interleukin-4 (IL-4, CM005-5MP, CHAMOT) and 20 ng/mL of interleukin IL-13 (IL-13, CM036-5MP, CHAMOT) for 48 h. All cells were cultured in RPMI-1640 media (HyClone) supplemented with 10% fetal bovine serum (FBS, Sigma-Aldrich), 100 U/mL penicillin, and 100 µg/mL streptomycin (all from Gibco-Invitrogen) at 37 °C, in a 5% CO_2_ humidified incubator.

### Animals

Female 6- to 8-week-old wild-type BALB/c mice or BALB/c nude mice were purchased from GemPharmatech (Nanjing, China) and bred in our animal facility under specific-pathogen-free conditions with fresh water and rodent diet available at all times. All animal procedures were carried out under protocols that complied with the Institutional Animal Care and Use Committee Guidelines for Ethical Conduct in the Care and Use of Animals.

### Characterization of the in vitro and in vivo photothermal behaviors of CNH-PG

For characterization of the photothermal behavior of CNH-PG in vitro, 0.5 mL of CNH-PG working solution of different concentrations (0, 5, 10, 20, 40 μg/mL) placed in a 1.5 mL EP tube was irradiated with 808 nm laser at a power density of 1 W/cm^2^ within a light spot 1 cm in diameter for 10 min. In addition, 0.5 mL of CNH-PG (10 μg/mL) placed in a 1.5 mL EP tube was irradiated with 808 nm laser of different power densities (0.8 W/cm^2^, 1 W/cm^2^, 1.4 W/cm^2^) within a light spot 1 cm in diameter for 10 min. The photothermal stability of CNH-PG at a concentration of 10 μg/mL was also measured. The CNH-PG aqueous solution was heated to 47 °C with 808 nm laser irradiation (LI) at 1.5 W/cm^2^ within a light spot 1 cm in diameter for 3 min and then allowed to cool down to room temperature. The cycle of heating and cooling was repeated five times. An infrared thermal camera (FLIRONE) was used to record the temperature changes and acquire thermal images. To explore the light irradiation conditions used in the in vitro cell experiments, 0.2, 0.5, or 1 mL of CNH-PG at a concentration of 10 μg/mL placed in 48, 24, or 12-well plates respectively were irradiated with 808 nm laser of different power densities (1.1 W/cm^2^, 1.3 W/cm^2^, 1.5 W/cm^2^, and 1.7 W/cm^2^) within a light spot 2 cm in diameter for 10 min while the area outside the well under LI was covered with tin foil, and the temperature of the solution was measured and recorded with an electronic thermometer in real-time. A FLIRONE was used to record the temperature changes and acquire thermal images. The photothermal behavior of CNH-PG in vivo was characterized as described below. Subcutaneous 4T1 graft tumors were established by inoculating 1 × 10^6^ 4T1 cells in 100 μL of PBS into the right flank of a BALB/c mouse or BALB/c nude mouse under anesthesia. Once the tumor volume reached 100 ~ 200 mm^3^, about 20 μL of CNH-PG solution (5 mg/mL) at a dosage of 5 mg/kg body weight was injected directly into the tumor. The tumor was then irradiated with an 808 nm laser (0.51 W/cm^2^ or 0.78 W/cm^2^, a light spot 1 cm in diameter) for 10 min at 4 h post-injection. Thermal images of the whole mouse were acquired at different time points with an infrared thermal camera (Additional file 1: Fig. S1D, E, G). As a control, some tumor-bearing mice received intratumoral injections of PBS (20 μL).

### Anti-tumor study on in vivo 4T1 tumor models

To establish graft tumors, 1 × 10^6^ 4T1 cells suspended in 100 μL of PBS were subcutaneously injected into the right flank of a female BALB/c mouse. When subcutaneous tumors were palpable, the animals were randomly divided into 8 groups, 5 mice per group, and subjected to treatments described in Table 1. The treatments were repeated every four days for a total of three times (on days 0, 4, 8). The tumor volume and body weight of each mouse were recorded from day 0 until the end of the experiment. The tumor volume was calculated according to the following formula: width^2^ × length × 0.5. On day 12 post the first administration, the tumor nodules were collected and subjected to relevant analyses after weight and size were taken. To explore the role of TAMs in the anti-tumor therapeutic efficacy, tumor cell inoculation was performed as described above and half of the animals (Mφ -) were randomly taken out for macrophage depletion when the tumor volume reached 100 ~ 200 mm^3^_._ Macrophage depletion was achieved through injection of liposome chlorophosphite (LIPOSOMA, 5 mg/mL, 200 μL/per mouse) by tail vein every other day. The other half of the animals (Mφ +) were each injected with an equal volume of PBS every other day. Six tumor-bearing mice, three from Mφ− and three from Mφ + , were sacrificed for determination of macrophage abundance in the tumor by immunofluorescent and immunohistochemical staining of F4/80 and CD11b at 48 h post the first injection of LIPOSOMA (Additional file 1: Fig. S1A–C). Twenty-four hours after the first LIPOSOMA or PBS injection, both the Mφ− and Mφ + mice were then each divided into six groups (6 mice per group) and subjected to treatments described in Table 2. The treatments were repeated every four days for a total of three times. The tumor volume and body weight of each mouse were recorded from the day of the first treatment until the end of the experiment. On day 13 post the first administration, the tumor nodules were collected and subjected to relevant analyses after weight and size were taken. The expression of PD-L1 was determined by immunofluorescent and immunohistochemical (IHC) staining.

**Table 1:** Animal grouping and treatments for in vivo anti-tumor study

	Treatments	Groups							
		①	②	③	④	⑤	⑥	⑦	⑧
CNH-PG	(5 mg/mL, 20 μL per mouse)		+	+	+		+	+	+
BMS-1	(2.5 mg/kg body weight)					+	+	+	+
LI	(808 nm, 0.51 W/cm^2^, 10 min)			+				+	
	(808 nm, 0.78 W/cm^2^, 10 min)				+				+
PBS	(20 μL per mouse)	+							

CNH-PG was given via intratumoral injection and BMS-1 was given via intraperitoneal injection. LI was applied at 4 h after CNH-PG injection

**Table 2:** Animal grouping and treatments for in vivo study exploring the role of TAMs in the anti-tumor therapeutic efficacy

	Treatments	Groups (Mφ + / Mφ−)					
		①	②	③	④	⑤	⑥
CNH-PG	(5 mg/mL, 20 μL per mouse)		+	+		+	+
BMS-1	(2.5 mg/kg body weight)				+	+	+
LI	(808 nm, 0.78 W/cm^2^, 10 min)			+			+
PBS	(20 μL per mouse)	+					

CNH-PG was given via intratumoral injection and BMS-1 was given via intraperitoneal injection. LI was applied at 4 h after CNH-PG injection

### IHC analysis

Antibodies for immunohistochemical analysis included PCNA (ab92552, Abcam), Ki67 (ab16667, Abcam), cle-caspase 3 (9664, CST), PD-L1 (66248-1-Ig, Proteintech), CD206 (ab64693, Abcam), CD80 (BS-2211R, BIOSS), CD86 (ab213044, Abcam), GBP5 (132201-AP, Proteintech), iNOS (ab283655, Abcam), ARG1 (GB11285, Servicebio), CD11b (ab133357, Abcam) and F4/80 (ab300421, Abcam). Paraffin sections (5 μm) were dewaxed, rehydrated, subjected to antigen repair with sodium citrate for 20 min, and then incubated in 3% hydrogen peroxide for 10 min at room temperature. The paraffin sections were then blocked with 5% BSA for 30 min, stained with antibodies overnight at 4 °C, washed with PBS, and stained with a secondary antibody (SV0004, BOSTER) for 1 h at room temperature. Diaminobenzidine (DAB, AR1025, BOSTER) was applied for coloration for 5 min at room temperature. Hematoxylin was used to stain the nucleus.

### Western blotting

Cells subjected to required treatments in six-well plates were rinsed twice with ice-cold PBS and lysed in RIPA buffer with a 1% protease inhibitor cocktail. Cell lysates were cleared by centrifugation and protein concentration was determined with a bicinchoninic acid (BCA) assay kit (Biosharp, BL521A). Equal protein aliquots (25 μg) were fractionated by SDS-PAGE and transferred to a PVDF membrane. The membranes were blocked with 5% fat-free milk in TBST and incubated with antibodies against Caspase-3 (19677-1-AP, Proteintech), cle-caspase 3 (9664, CST), PD-L1 (66248-1-Ig, Proteintech), BCL2 (26593-1-AP, Proteintech), BAX (50599-2-Ig, Proteintech), GBP5 (132201-AP, Proteintech), iNOS (ab283655, Abcam), ARG1 (GB11285, Servicebio) and GADPH (BioPM, PMK053) overnight at 4 °C. Protein bands were imaged using a horseradish peroxidase-conjugated secondary antibody and ECL and the films were exposed with a Bio Imaging system (Syngene).

### Immunofluorescent staining of tissue sections

For fluorescent immunostaining of the tumor tissue, paraffin sections (5 μm) were dewaxed and rehydrated, subjected to antigen repair with sodium citrate for 20 min, and then blocked with 5% BSA for 30 min at room temperature. The paraffin sections were then stained with antibodies against F4/80 (70076, CST) overnight at 4 °C, washed with PBS, and stained with an anti-rabbit secondary antibody (SV0004, BOSTER) for 1 h at room temperature. Next, the paraffin sections were stained with CY3-labeled tyramine salt for 20 min at room temperature, washed with PBS, subjected to antigen repair, and blocked again. Finally, the paraffin sections were stained with antibodies against PD-L1 (66248-1-Ig, Proteintech), CD86 (ab213044, Abcam), and CD206 (ab64693, Abcam) overnight at 4 °C, stained with secondary antibody (SV0004, BOSTER) for 50 min and stained with AF488 labeled tyramine salt for 20 min at room temperature. Paraffin sections were then examined under a microscope (OLYMPUS, Japan).

### Immunofluorescent staining of cells and flow cytometry

Cells to be analyzed were washed and resuspended in PBS, followed by incubation with antibodies for PD-L1 (66248-1-Ig, Proteintech), CD206 (ab64693, Abcam), CD80 (BS-2211R, BIOSS), CD86 (ab213044, Abcam), APC anti-mouse F4/80 (157305, Biolegend), FITC anti-mouse F4/80 (157309, Biolegend), APC anti-mouse CD11b (M1/70, APC-65055, Proteintech) and APC-conjugated secondary antibody (Bioss, bs-0368G-APC/bs-0295G-APC). After incubation for 30 min at 4 °C, PBS was used to wash the cells. Then cellular fluorescence was acquired on a Beckman Cytoflex flow cytometer (CA, USA). Histogram geometric means (GM) were used to quantify the mean fluorescent intensity (MFI).

### Cell viability assay

(1) 4T1 cells were treated with CNH-PG at concentrations up to 100 μg/mL for 24 h, then cell viability was assayed. (2) 4T1 cells were treated with or without 10 μg/mL CNH-PG for 30 min, then irradiated with 808 nm laser (1.3 W/cm^2^ or 1.5 W/cm^2^) for 10 min, and then assayed for viability after 24 h of continued incubation. (3) The 4T1 or BMDM2 cells were pretreated with 10 μg/mL CNH-PG and BMS-1 at concentrations up to 10 μM for 30 min, then irradiated with 808 nm laser (1.3 W/cm^2^ or 1.5 W/cm^2^) for 10 min, then assayed for viability after 24 h of continued incubation. Cell viability was assayed using a CCK-8 kit as instructed in the manual provided by the kit manufacturer (Dojindo Molecular Technologies, Inc., Japan).

### Assay of cell proliferation and death

For analysis of cell proliferation, 4T1 cells were labeled with 2 μM of 5(6)-carboxyfluorescein diacetate, succinimidyl ester (CFSE, Sigma-Aldrich, USA) according to a previously published protocol [45]. 4T1 cells with or without CFSE labeling in a single culture or in co-culture with BMDM2 were pretreated with 2 μM of BMS-1 and/or 10 μg/mL of CNH-PG for 30 min, then irradiated with 808 nm laser (1.3 W/cm^2^ or 1.5 W/cm^2^) for 10 min, The cells were then taken after 24 h for flow cytometry analysis of cell surface annexin v staining and decay of CFSE staining indicative of cell proliferation per a previously published protocol [46]. In addition, the protein levels of PD-L1, caspase 3, cleaved-caspase 3, BAX, BCL-2, γH2AX, and GAPDH were determined by western blotting, and the expression of PD-L1 on the cell surface was determined by immunofluorescent staining and flow cytometry.

### Macrophage phenotyping

The BMDM2 cells either in a single culture or mixed-cultured with 4T1 cells were pretreated with 2 μM of BMS-1 and/or 10 μg/mL CNH-PG for 30 min, then irradiated with 808 nm laser (1.3 W/cm^2^ or 1.5 W/cm^2^) for 10 min. The cells were taken out after 24 h and stained with antibodies for F4/80, CD80, CD86, and CD206 for flow cytometry assay. The protein levels of PD-L1, iNOS, GBP5, ARG1, and GAPDH were assayed by western blotting. In addition, the phagocytic function of the BMDM2 cells was determined by assaying the internalization of fluorescent latex beads (2 μm, blue, Sigma L0280). The beads which were opsonized by incubating with PBS supplemented with 50% FBS were subsequently added to the cells and incubated at 37 °C for 2 h. The cells were then washed with pre-cooled PBS and assayed via flow cytometry.

### Data analysis

All data are presented as mean ± SD. Comparison between groups for statistical significance was performed using the unpaired Student’s t-test. For the comparison of more groups, a one-way analysis of variance (ANOVA) followed by the Neuman-Keuls post hoc test was used.

## Results

### Characterization of CNH-PG-mPT in vitro and in vivo

**Fig. 1:**
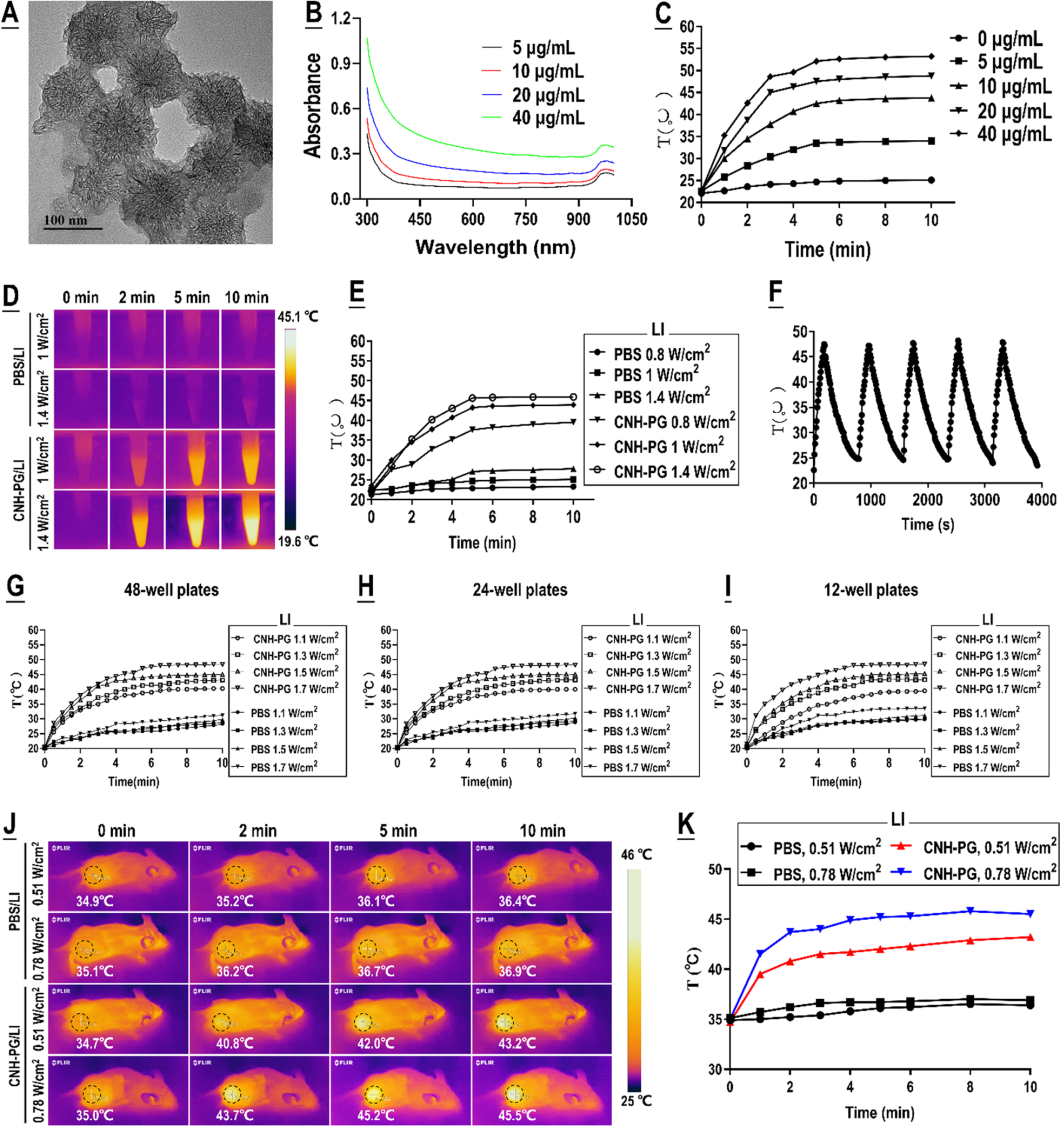
Characterization of CNH-PG-mediated photothermal conversion property. **A** TEM imaging of CNH-PG. **B** UV–vis spectroscopy of CNH-PG. **C** Temperatures obtained by 808 nm LI (1 W/cm^2^, a light spot 1 cm in diameter) on CNH-PG solutions of different concentrations. **D** Thermal imaging of 0.5 mL of CNH-PG (10 μg/mL) placed in a 1.5 mL EP tube under 808 nm LI (1 and 1.4 W/cm^2^, a light spot 1 cm in diameter) for different durations. **E** Temperatures obtained by 0.5 mL of CNH-PG (10 μg/mL) placed in a 1.5 mL EP tube under 808 nm LI (0.8, 1 and 1.4 W/cm^2^, a light spot 1 cm in diameter) for different durations. **F** Photothermal stability of CNH-PG. CNH-PG solution (10 μg/mL) was heated to 47 °C with 808 nm LI at 1.5 W/cm^2^ for 3 min (a light spot 1 cm in diameter) and then allowed to cool down to room temperature. The cycle of heating and cooling was repeated five times. **G**–**I** Temperatures obtained by 0.2, 0.5, or 1 mL of CNH-PG (10 μg/mL, in PBS) placed in a well of a 48-, 24-, or 12-well plate respectively under 808 nm LI (1.1, 1.3, 1.5 and 1.7 W/cm^2^, covered in a light spot 2 cm in diameter) for different durations. **J** Thermal imaging of subcutaneous 4T1 tumors injected with 20 μL of CNH-PG solution (5 mg/mL) that was subjected to 808 nm LI (0.51 or 0.78 W/cm^2^, a light spot 1 cm in diameter) for 10 min at 4 h post-injection. **K** Temperatures obtained in subcutaneous 4T1 tumors injected with 20 μL of CNH-PG solution (5 mg/mL) that was subjected to 808 nm LI (0.51 or 0.78 W/cm^2^, in a light spot 1 cm in diameter) for 10 min at 4 h post-injection

Figure 1A shows the morphology of the CNH-PG observed via TEM. Ultraviolet–visible-near infrared (UV–Vis-NIR) absorption spectroscopy of CNH-PG showed an absorption tail beyond 700 nm attributed to the strong NIR-absorption property of the CNH core (Fig. 1B). CNH-PG exhibited a narrow hydrodynamic size distribution, and the hydrodynamic diameter was 122 ± 18 nm in water. The hydrophilic PG layer afforded CNH-PG good aqueous dispersibility and stability. The concentration of CNH-PG water dispersion was no less than 10 mg/mL as measured by weighing the solid in the dispersion after evaporation of water. The hydrodynamic diameter of CNH-PG remained almost constant (126 ± 20 nm) over 30 days. CNH-PG possessed a negative surface charge of approximately -20 mV. Detailed information on the fabrication, chemical, and physical characterization of CNH-PG is provided in a previously published work [42]. Presented herein are mainly the characteristics of CNH-PG-mPT.

We first validated the CNH-PG-mPT, i.e. the photothermal conversion property of CNH-PG. As shown in Fig. 1C, continual LI (808 nm, 1 W/cm^2^) on 0.5 mL of CNH-PG water dispersion (5–40 μg/mL) placed in a 1.5 mL EP tube produced a steady elevation of temperature that plateaued at 5 min and held steady until 10 min when the LI was ceased, and the obtained plateau temperatures showed a dependence on CNH-PG concentration. The higher power of LI also resulted in higher plateau temperature which was 45 ℃ in the case of 1.4 W/cm^2^ of LI, in comparison with 43 ℃ at 1.0 W/cm^2^ of LI and 37 ℃ at 0.8 W/cm^2^ of LI (Fig. 1D, E). CNH-PG was able to generate the same level of temperature under repeated LI at intervals within a period of 4000 s, indicating a very stable photothermal conversion property (Fig. 1F). As shown in Fig. 1G–I, regardless of the size of the cell culture plates, irradiation of 10 μg/mL of CNH-PG with 808 nm LI in a light spot 2 cm in diameter with a power density of 1.3 W/cm^2^ and 1.5 W/cm^2^ could produce temperatures that plateaued at 43 ℃ and 45 ℃, respectively, at 5 min and held steady until 10 min when the LI was stopped. In vivo, mice bearing subcutaneous 4T1 graft tumors were intratumorally injected with 20 μL of CNH-PG dispersion in PBS (5 mg/mL) at a dosage of 5 mg/kg body weight. Four hours later when the injected CNH-PG had achieved distribution throughout the tumor tissue, the tumor received continual 808 nm LI with the tumor area covered by a light spot 1 cm in diameter. As shown in Fig. 1J, K, the tumor site under LI displayed a rapid temperature rise that plateaued at 3 min and held steady until 10 min when the LI ceased, and a higher LI power density led to a higher plateau temperature which was 43 ℃ at 0.51 W/cm^2^ and 45 ℃ at 0.78 W/cm^2^ of LI. By contrast, tumors injected with PBS exhibited insignificant temperature rise under LI.

Based on the above findings, the following conditions were adopted to generate a temperature of 43 or 45 ℃ in subsequent in vitro cell experiments: 10 min of continual LI in a light spot 2 cm in diameter with a power density of 1.3 W/cm^2^ or 1.5 W/cm^2^, applied to cells in the presence of 10 μg/mL of CNH-PG in welled plates; the area outside the wells was covered with tin foil. The conditions for generating a temperature of 43 °C or 45 ℃ in in vivo tumors were the following: 10 min of continual LI covering the tumor site in a light spot 1 cm in diameter with a power density of 0.51 W/cm^2^ or 0.78 W/cm^2^, applied 4 h after an intra-tumoral injection of 20 μL of CNH-PG (5 mg/mL in PBS) at a dosage of 5 mg/kg body weight.

### BMS-1 potentiated the anti-tumor efficacy of CNH-PG-mPT with a synergy

**Fig. 2:**
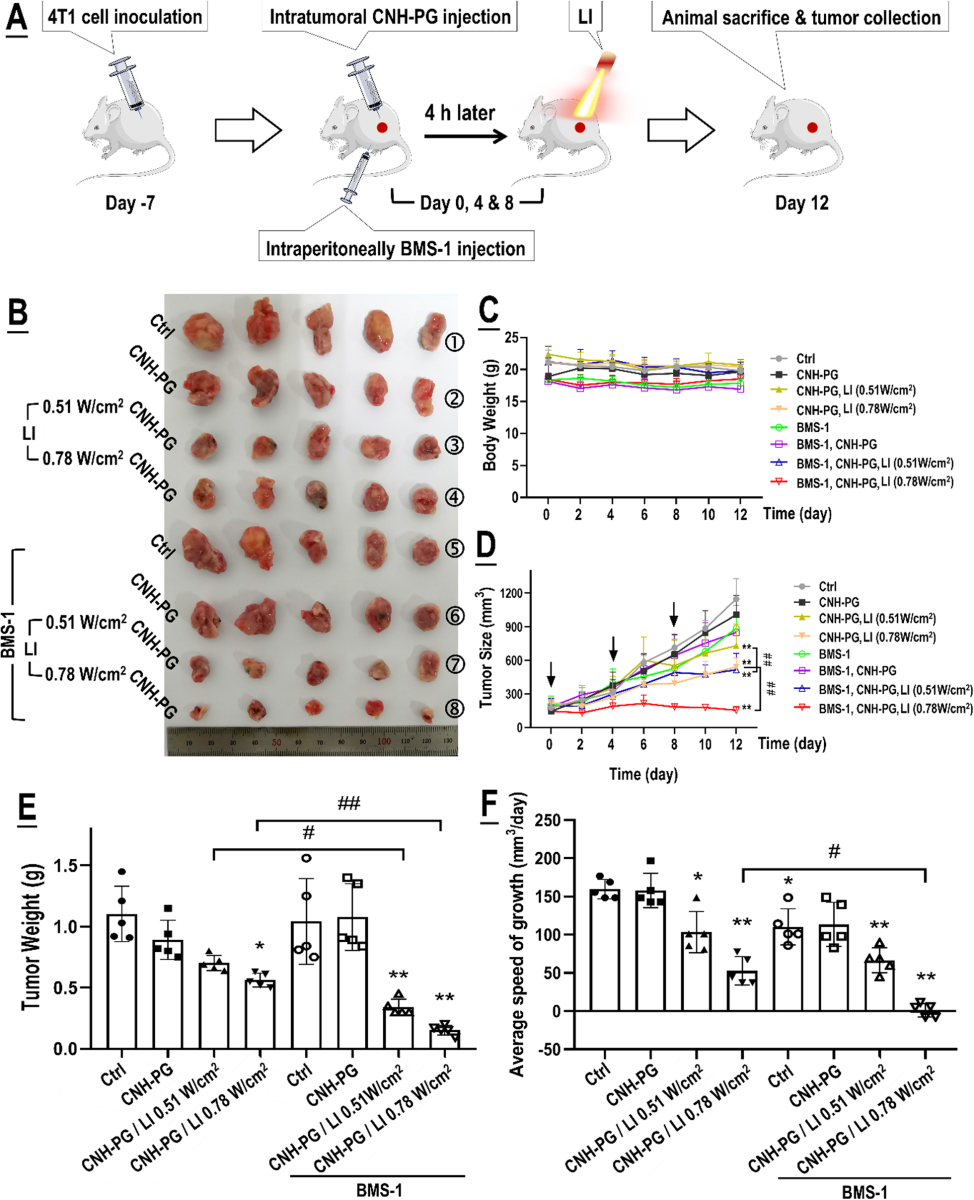
BMS-1 potentiated the anti-tumor therapeutic efficacy of CNH-PG-mPT in mice with a synergy. See Table 1 for animal grouping and treatments. **A** Illustration of the experimental procedure. **B** Graft tumors collected at the end of therapy. **C** Changes in animal body weight over the therapeutic period. **D** Tumor growth over the therapeutic period. **E** Tumor weights taken at the end of therapy. **F** Average rates of tumor growth. Values are means ± standard deviation (SD). (n = 5, ^# &^ *p < 0.05, ^## &^ **p < 0.01)

**Fig. 3:**
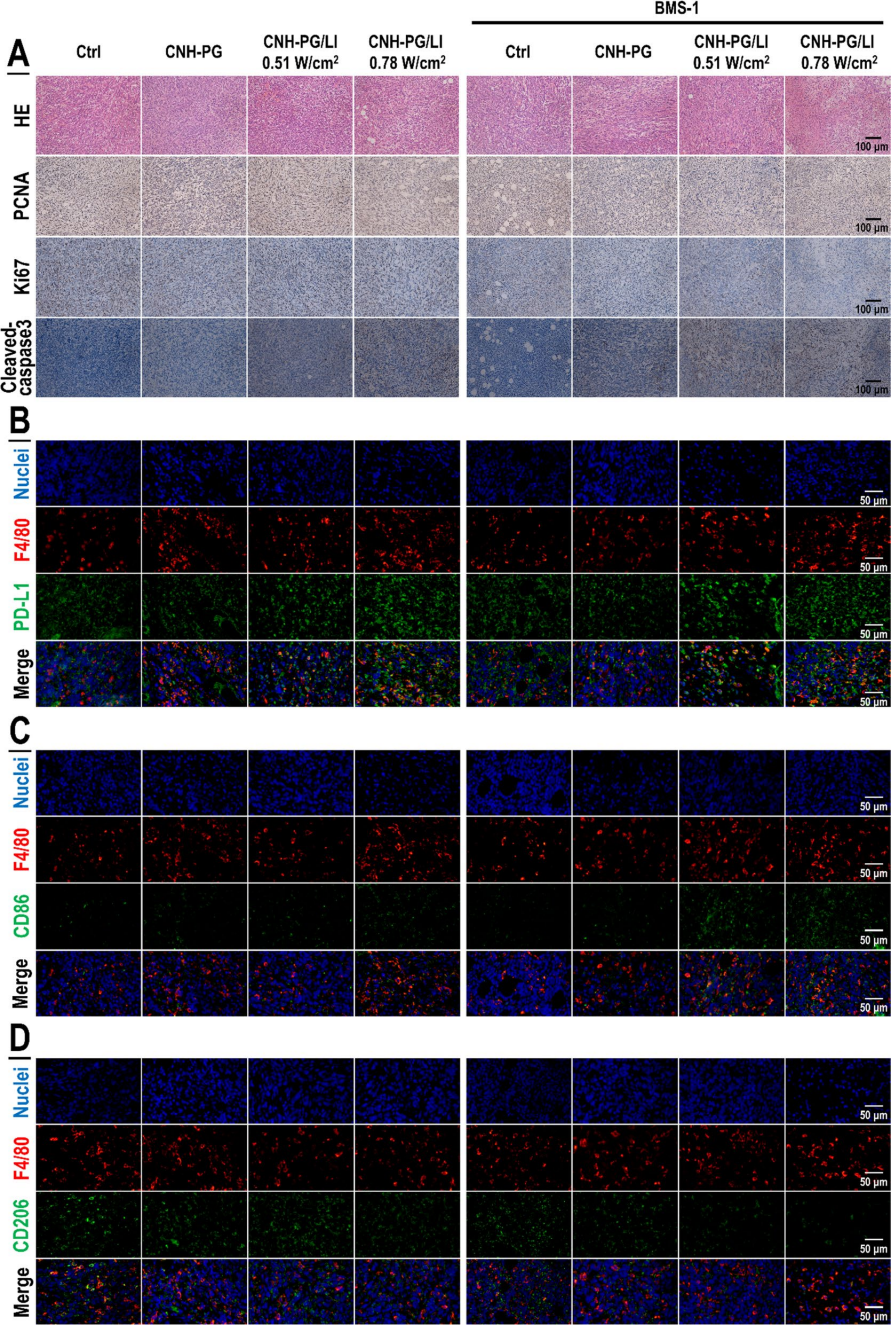
CNH-PG-mPT suppressed tumor cell growth and increased the presence of M1-like macrophages and PD-L1 expression in the tumor tissue and these effects (but for PD-L1 induction) were significantly enhanced by BMS-1. **A** Tumor tissue sections showing HE staining and IHC staining of PCNA, Ki67 (markers of cell growth), and cleaved caspase 3 (marker of cell death). Staining of PCNA, Ki67 and cleaved caspase 3 appeared as dark brown spots. **B** Tumor tissue sections showing immunofluorescent staining of F4/80 (a marker of macrophage) and PD-L1. The PD-L1 staining showed a more extensive distribution than the F4/80 staining and partial overlapping could be observed between the PD-L1 staining and the F4/80 staining. **C** Tumor tissue sections showing immunofluorescent staining of F4/80 and CD86 (a marker of M1-like activation). The F4/80 staining showed a more extensive distribution than the CD86 staining and partial overlapping could be observed between the F4/80 staining and the CD86 staining. **D** Tumor tissue sections showing immunofluorescent staining of F4/80 and CD206 (a marker of M2-like activation). The F4/80 staining showed a more extensive distribution than the CD206 staining and partial overlapping could be observed between the F4/80 staining and the CD206 staining. The quantitative analysis results of IHC in **A** and IF staining in **B**–**D** are presented in Additional file 1: Fig. S2

We next looked into the synergistic therapeutic efficacy of CNH-PG-mPT and BMS-1 against mouse TNBC. The treatment protocol was present in Fig. 2A. Briefly, for CNH-PG-mPT, each subcutaneous tumor received one injection of CNH-PG and post-injection LI at the tumor site on days 0, 4, and 8. For combinatorial therapy, part of the mice that received CNH-PG-mPT also received a dose of BMS-1 (2.5 mg/kg body weight) via intraperitoneal injection on the same day of CNH-PG-mPT. Endpoint tumor size and weight (Fig. 2B, D, E), and particularly tumor growth (Fig. 2D, F) all indicated a pronounced synergy of CNH-PG-mPT (induced both by 0.51 W/cm^2^ and 0.78 W/cm^2^ of LI) and BMS-1 in achieving anti-TNBC efficacy. Of note, CNH-PG-mPT, particularly produced by 0.78 W/cm^2^ of LI, appeared to show some therapeutic efficacy while the therapeutic efficacy of BMS-1 alone or plus CNH-PG was unremarkable. As further substantiation to the anti-tumor synergy of CNH-PG-mPT and BMS-1, histological examination revealed massive downregulation of PCNA and Ki67 expression, and upregulated expression of cleaved caspase 3 in tumors that received CNH-PG-mPT plus BMS-1, indicating occurrence of significant growth suppression and cell death (Fig. 3A). Importantly, markedly increased PD-L1 expression was observed in tumors that received CNH-PG-mPT, irrespective of BMS-1 (Fig. 3B). Meanwhile, CNH-PG-mPT (particularly produced by 0.78 W/cm^2^ of LI) was found to increase the presence of macrophages in the tumors and the macrophages appeared to be an M1-like phenotype, as indicated by the increased staining of F4/80 and CD86, and decreased staining of CD206 (Fig. 3C, D). Notably, this effect was markedly enhanced by BMS-1, particularly in the case of CNH-PG-mPT produced by 0.78 W/cm^2^ of LI (Fig. 3C, D). One more observation worth particular noting is the PD-L1 staining which was more extensive than the staining of macrophage markers across all groups indicating PD-L1 expression both in the tumor cells and the macrophages (Fig. 3B–D). Additionally, all treated mice varied little from control in body weight indicating the absence of systemic toxicity (Fig. 2C).

Taken together, the above findings suggest that (1) CNH-PG-mPT has a low level of anti-TNBC efficacy; (2) CNH-PG-mPT increases the presence of M1-like macrophages and PD-L1 expression in the tumor tissue; (3) these effects (but for PD-L1 induction) of CNH-PG-mPT can be significantly enhanced by BMS-1; (4) both the tumor cells and the infiltrating macrophages express PD-L1.

### BMS-1 potentiated CNH-PG-mPT’s toxicity to tumor cells

**Fig. 4:**
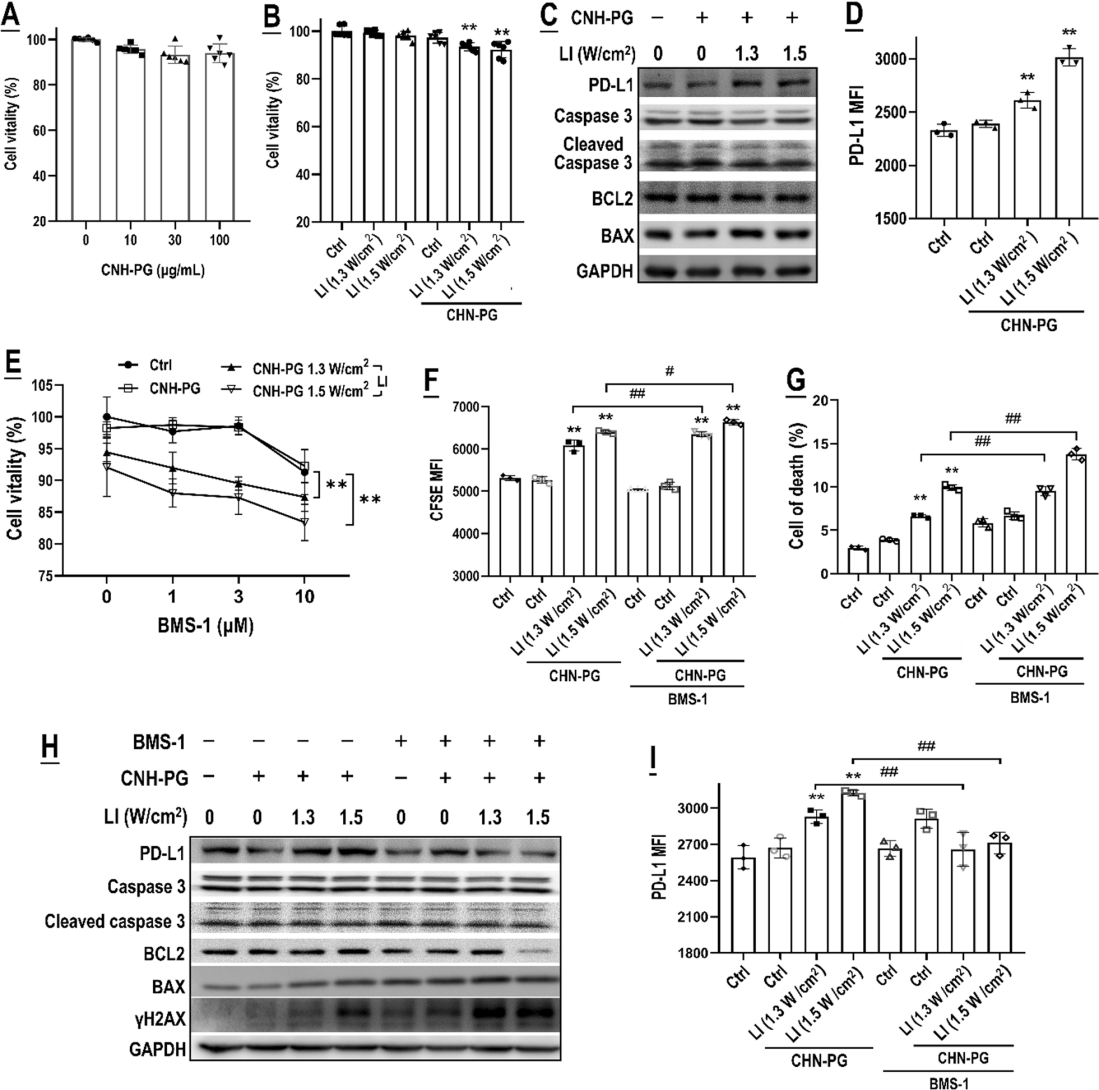
BMS-1 potentiated CNH-PG-mPT’s toxicity to tumor cells. **A** Effect of CNH-PG on 4T1 cell viability. **B** Effects of LI and CNH-PG-mPT on 4T1 cell viability. **C** Effects of CNH-PG and CNH-PG-mPT on the protein levels of PD-L1, caspase 3, BCL2, and BAX. The gray analysis results of caspase 3, BCL2, and BAX are presented in Additional file 1: Fig. S3. **D** Effects of CNH-PG and CNH-PG-mPT on 4T1 cell surface PD-L1 abundance. **E** Effects of CNH-PG-mPT, BMS-1, and their combination on 4T1 cell viability. Effects of CNH-PG, BMS-1, CNH-PG plus BMS-1, CNH-PG-mPT, and CNH-PG-mPT plus BMS-1 on **F**, **G** 4T1 cell proliferation and death; **H** protein levels of PD-L1, caspase 3, BCL2, and BAX; and **I** 4T1 cell surface PD-L1 abundance. Cell viability was assayed using a CCK-8 kit. Protein levels were assayed by western blotting. Cell surface PD-L1 abundance was assayed via immunofluorescent staining and flow cytometry. Cell proliferation was assayed via CFSE staining and flow cytometry. Cell death was assayed via annexin V staining and flow cytometry. Values are means ± standard deviation (SD). (n = 3, # & *p < 0.05, ## & **p < 0.01). Representative flow cytometry dot plots/histograms are provided in Additional file 1: Fig. S4

In vitro experiments were then conducted to verify the above-raised suggestions. Cell viability assay showed that neither CNH-PG (up to 100 μg/mL) nor LI (1.3 and 1.5 W/cm^2^, 10 min) was toxic to the 4T1 cells, but CNH-PG-mPT (10 μg/mL of CNH-PG plus LI) exhibited a low level of cytotoxicity (Fig. 4A, B). This is in line with the expression levels of cell death/survival markers (cleaved caspase 3, BAX & BCL2) that varied little from control (Fig. 4C). Notably, CNH-PG-mPT (10 μg/mL of CNH-PG plus LI) resulted in marked PD-L1 upregulation in the tumor cells (Fig. 4C, D). The observations aligned well with the in vivo findings on PD-L1 presented in Fig. 3B. Neither BMS-1 alone nor in combination with CNH-PG (10 μg/mL) was toxic to the 4T1 cells when its concentration was not above 3 μM (Fig. 4E). However, once LI was involved i.e. once BMS-1 was used in combination with CNH-PG-mPT, cytotoxicity began to show and became more pronounced with increasing concentrations of BMS-1 (Fig. 4E). Consistently, 2 μM of BMS-1 in combination with CNH-PG-mPT resulted in significant cytotoxicity as evidenced by increased staining of CFSE and annexin v (indicating growth suppression and apoptosis) (Fig. 4F, G), elevated expression of γH2X and cleaved caspase 3 & BAX (indicating DNA damage and cell death), and downregulation of BCL 2 (marker of cell survival) (Fig. 4H). It is worth noting that BMS-1 appeared to be able to suppress the PD-L1 upregulation induced by CNH-PG-mPT as indicated by a decrease both in total PD-L1 protein level and cell surface PD-L1 abundance (Fig. 4H, I).

### BMS-1 potentiated CNH-PG-mPT to skew TAMs into an anti-tumor M1-like phenotype

**Fig. 5:**
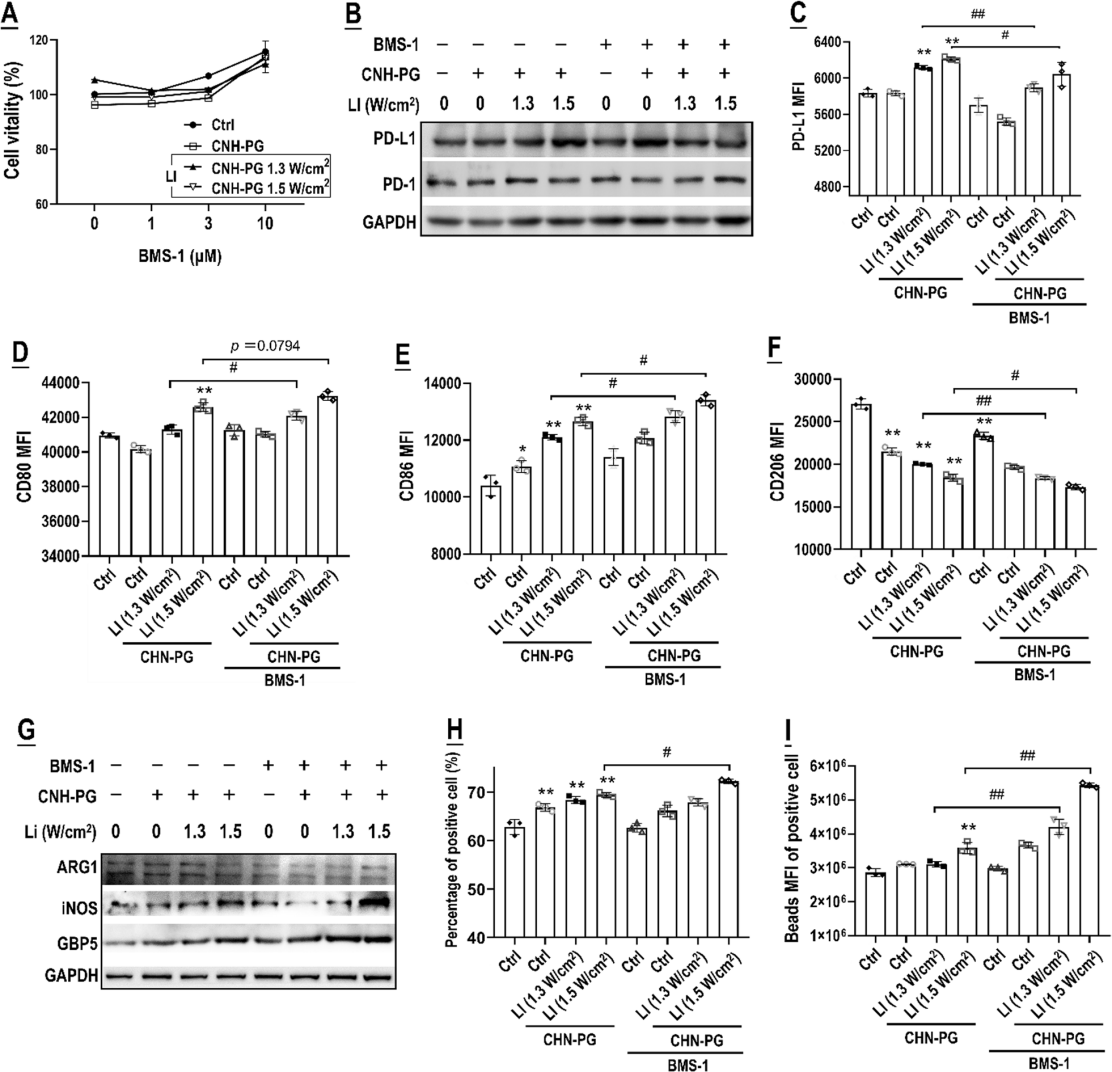
BMS-1 potentiated CNH-PG-mPT to skew TAMs into an M1-like phenotype. **A** Effects of CNH-PG and CNH-PG-mPT on M2 cell viability. Effects of CNH-PG, BMS-1, CNH-PG plus BMS-1, CNH-PG-mPT, and CNH-PG-mPT plus BMS-1 on **B** protein content of PD-L1 and PD-1 in M2; **C** cell surface PD-L1 abundance in M2. The gray analysis result of PD-1 is presented in Additional file 1: Fig. S3; **D**–**F** cell surface abundance of CD80, CD86, and CD206 in M2; **G** protein content of ARG1, iNOS, and GBP5; and **H**, **I** phagocytic activity of M2. Cell viability was assayed using a CCK-8 kit. Protein levels were assayed by western blotting. Cell surface protein abundance was assayed via immunofluorescent staining and flow cytometry. The phagocytic function was assayed using fluorescent latex beads and flow cytometry. Cell death was assayed via annexin V staining and flow cytometry. Values are means ± standard deviation (SD). (n = 3, # & *p < 0.05, ## & **p < 0.01). Representative flow cytometry dot plots/histograms are provided in Additional file 1: Fig. S5

**Fig. 6:**
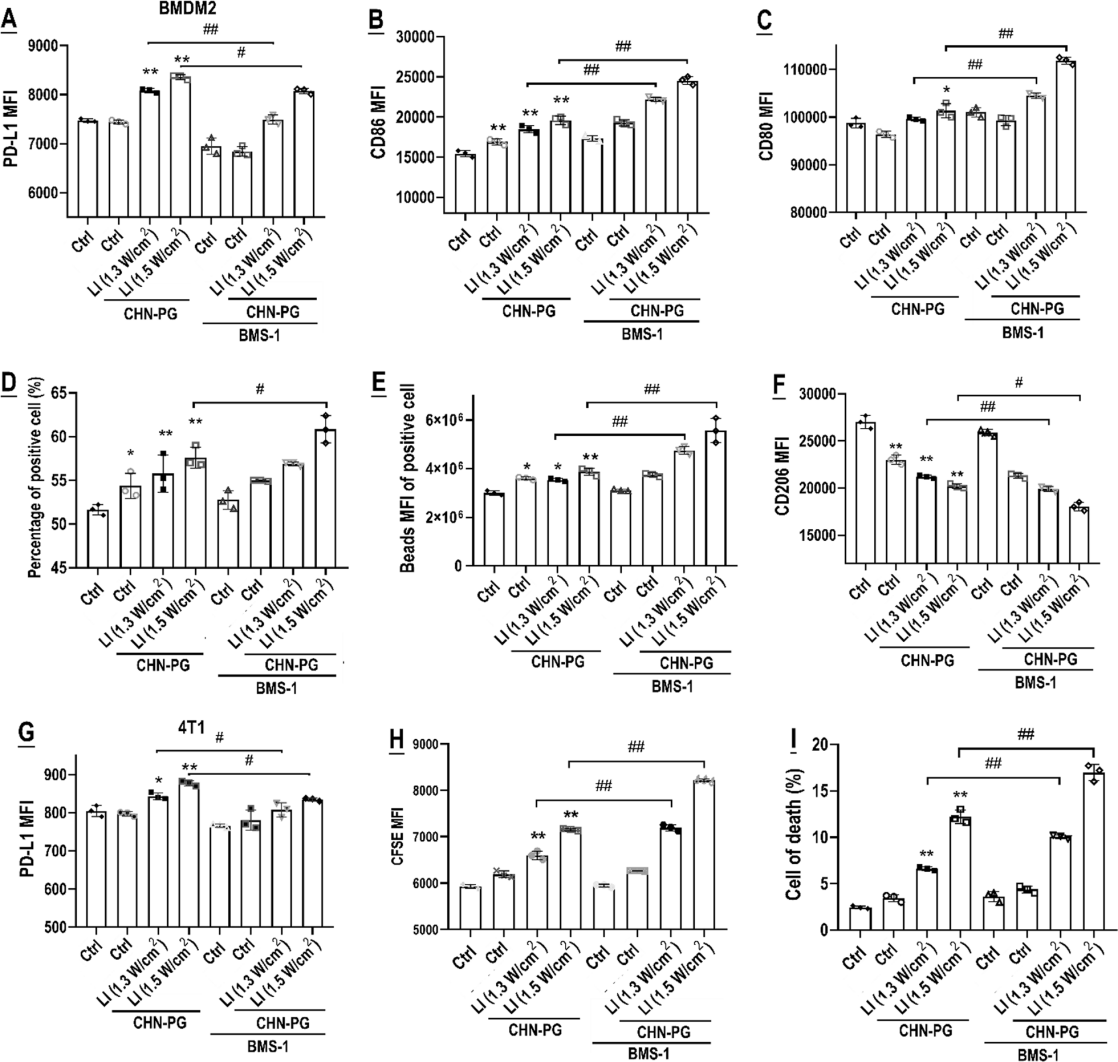
BMS-1 synergized with CNH-PG-mPT to skew TAMs in co-culture with 4T1 cells into an anti-tumor M1-like phenotype. Effects of CNH-PG, BMS-1, CNH-PG plus BMS-1, CNH-PG-mPT, and CNH-PG-mPT plus BMS-1 on **A**–**C** and **F** cell surface abundance of PD-L1, CD86, CD80, and CD206 in M2; and **D**, **E** phagocytic activity of M2. **G** Cell surface abundance of PD-L1 in co-cultured 4T1 cells. **H**, **I** Proliferation and death of co-cultured 4T1 cells. Cell surface protein abundance was assayed via immunofluorescent staining and flow cytometry. The phagocytic function was assayed using fluorescent latex beads and flow cytometry. Cell proliferation was assayed via CFSE staining and flow cytometry. Cell death was assayed via annexin V staining and flow cytometry. Values are means ± standard deviation (SD). (n = 3, # & *p < 0.05, ## & **p < 0.01). Representative flow cytometry dot plots/histograms are provided in Additional file 1: Fig. S6. Normalized data of TAM phenotyping and 4T1 toxicity from Figs. 4, 5, 6 showing the synergy of BMS-1 with CNH-PG-mPT in single culture and co-culture are presented in Additional file 1: Table S1 for comparison

On the side of TAMs, BMS-1 (up to 10 μM) did not show apparent toxicity to TAMs either alone or in the presence of CNH-PG (10 μg/mL) or CNH-PG-mPT (10 μg/mL plus 10 min of 808 nm LI) as indicated by cell viability assay (Fig. 5A). Interestingly, as in the case of the 4T1 cells (Fig. 4I), CNH-PG-mPT effected a marked increase of PD-L1 expression both in total protein and cell surface abundance, and this PD-L1 upregulation could be repressed by BMS-1 (2 μM) (Fig. 5B, C). Notably, CNH-PG-mPT also increased PD-1 expression in the TAMs (Fig. 5B). Next, the TAMs phenotype was examined after treatment of CNH-PG-mPT or CNH-PG-mPT plus BMS-1. As shown in Fig. 5D–I, CNH-PG-mPT skewed the TAMs towards an M1-like phenotype as indicated by the marked upregulation of CD80, CD86, iNOS, GBP5, and phagocytic function, but downregulation of CD206 and ARG1, and BMS-1 gave a remarkable boost to CNH-PG-mPT to induce the M1-like phenotype. The same pattern of phenotypic shifts was observed in the TAMs in co-culture with 4T1 cells when the co-culture had been subjected to CNH-PG-mPT or CNH-PG-mPT plus BMS-1 (Fig. 6A–F). Briefly, the TAMs exhibited upregulated surface PD-L1, M1-like surface markers e.g. CD86 & CD80, and phagocytic function, but downregulated CD206 and these changes (but for the PD-L1 upregulation) were more pronounced under CNH-PG-mPT plus BMS-1 than under CNH-PG-mPT alone. On the other hand, the 4T1 cells in the co-culture, like their single-cultured counterparts, also displayed PD-L1 upregulation, growth suppression, and cell death under CNH-PG-mPT and these responses (but for PD-L1 upregulation) were more pronounced under CNH-PG-mPT plus BMS-1 (Fig. 6G–I). Like in single culture, BMS-1 repressed CNH-PG-mPT-induced PD-L1 upregulation in co-cultured 4T1 cells and TAMs (Fig. 6A, G).

 Importantly, a review juxtaposing Figs. 4, 5, and 6 reveals that the synergistic effects of CNH-PG-mPT and BMS-1 both on the TAMs (M1-like activation) and the 4T1 cells (toxicity) were more striking when the two cell populations were in co-culture (Additional file 1: Table S1). An element of tumor cell-TAMs interaction is thus suggested in the synergy of CNH-PG-mPT and BMS-1 when the two cell populations are in each other’s presence.

### The anti-tumor efficacy of CNH-PG-mPT alone and in synergy with BMS-1 were both dependent on TAMs

**Fig. 7:**
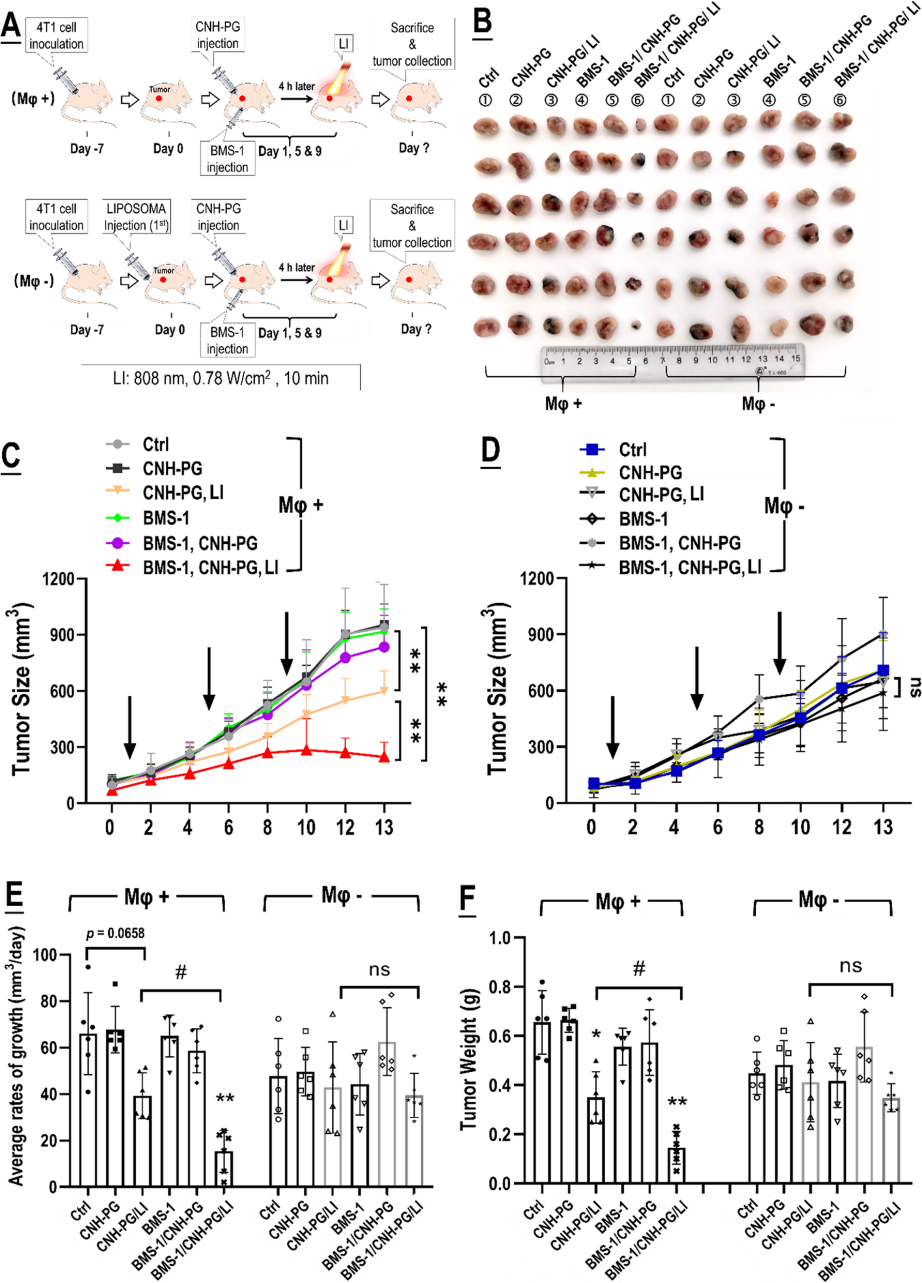
TAMs depletion largely abolished the synergetic anti-tumor efficacy of CNH-PG-mPT and BMS-1. See Table 2 for animal grouping and treatments. **A** Illustration of the experimental procedure. **B** Graft tumors collected at the end of therapy. **C**, **D** Tumor growth over the therapeutic period. **E** Average rates of tumor growth. **F** Tumor weights taken at the end of therapy. **F** Mφ + indicates mice without macrophage depletion; Mφ− indicates mice depleted of macrophages. Values are means ± standard deviation (SD). (n = 6, ^#,&,^*p < 0.05, ^##,&,^**p < 0.01)

**Fig. 8:**
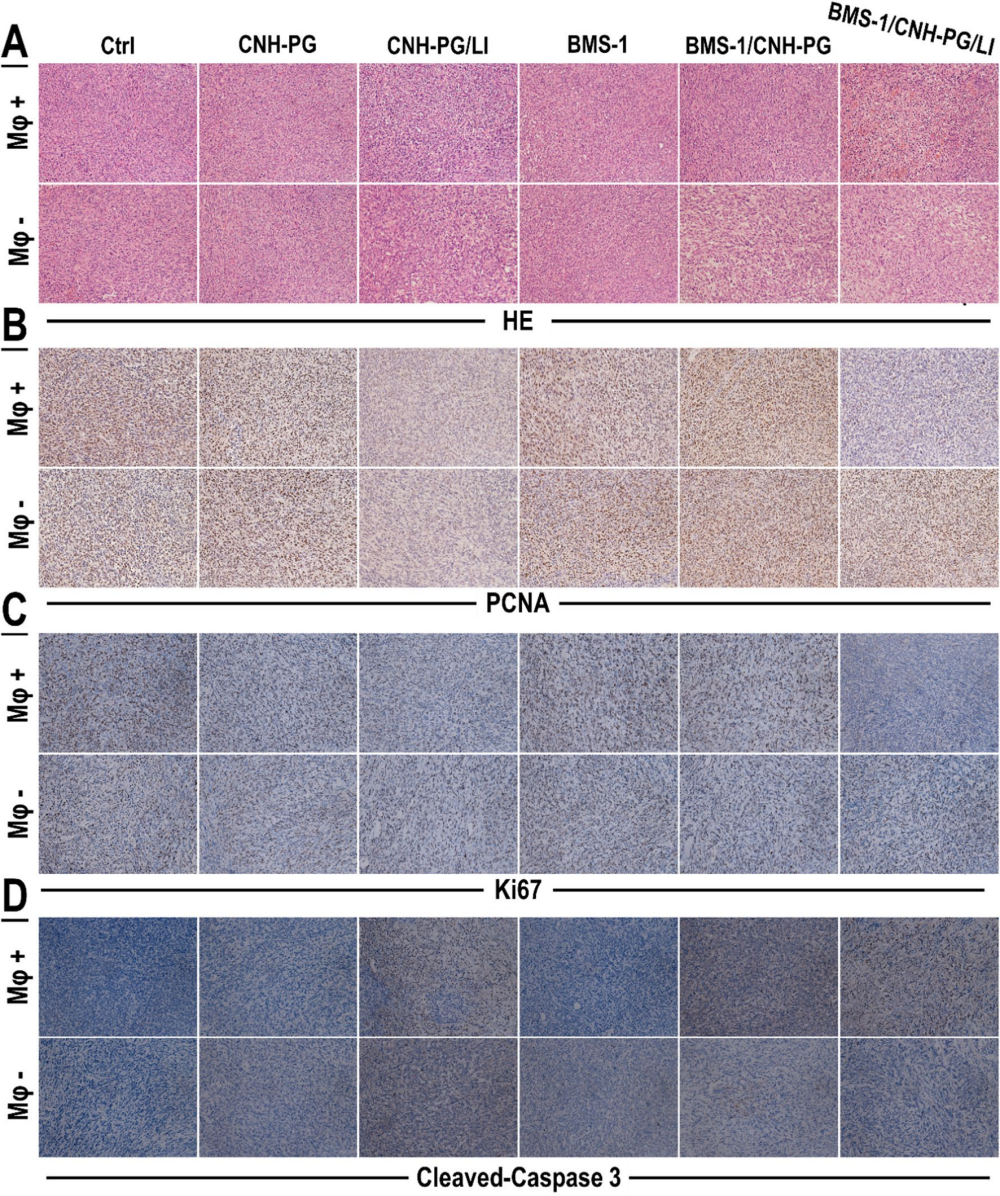
Cell proliferation and death profiles in the tumors with or without TAMs depletion. **A** Tumor tissue sections showing HE staining. **B**–**D** Tumor tissue sections showing IHC staining of PCNA and Ki67 (markers of cell growth) and cleaved caspase 3 (marker of cell death). Staining of PCNA, Ki67 and cleaved caspase 3 appeared as dark brown spots. Mφ + indicates mice without macrophage depletion; Mφ− indicates mice depleted of macrophages

 In vivo experiments adopting TAMs depletion were next conducted to explore the role of tumor cell-TAMs interaction in, or more specifically, the contribution of TAMs to the synergistic anti-TNBC efficacy of CNH-PG-mPT and BMS-1. Figure 7A shows the experiment procedure. As shown in Fig. 7B–F, 4T1 tumors depleted of TAMs exhibited slower growth, lower endpoint size, and weight than their counterparts without TAMs depletion, indicating a pro-tumor role of macrophages. Similar to the observations presented in Figs. 2 and 3A, CNH-PG-mPT showed some degree of therapeutic efficacy in 4T1 tumors without TAMs depletion, which was massively potentiated by tandem use of BMS-1 (Fig. 7B, C, E, F). In striking contrast, CNH-PG-mPT lost its therapeutic efficacy not only as a single therapy but also in combination with BMS-1 in 4T1 tumors with depleted TAMs (Fig. 7B–F). IHC staining of tumor tissues yielded consistent observations indicating repressed tumor growth and increased cell death under CNH-PG-mPT, which were aggravated by BMS-1 (Fig. 8), in 4T1 tumors not depleted of TAMs. The above findings strongly suggest that the therapeutic efficacy of CNH-PG-mPT alone and in combination with BMS-1 are both heavily dependent on TAMs.

**Fig. 9:**
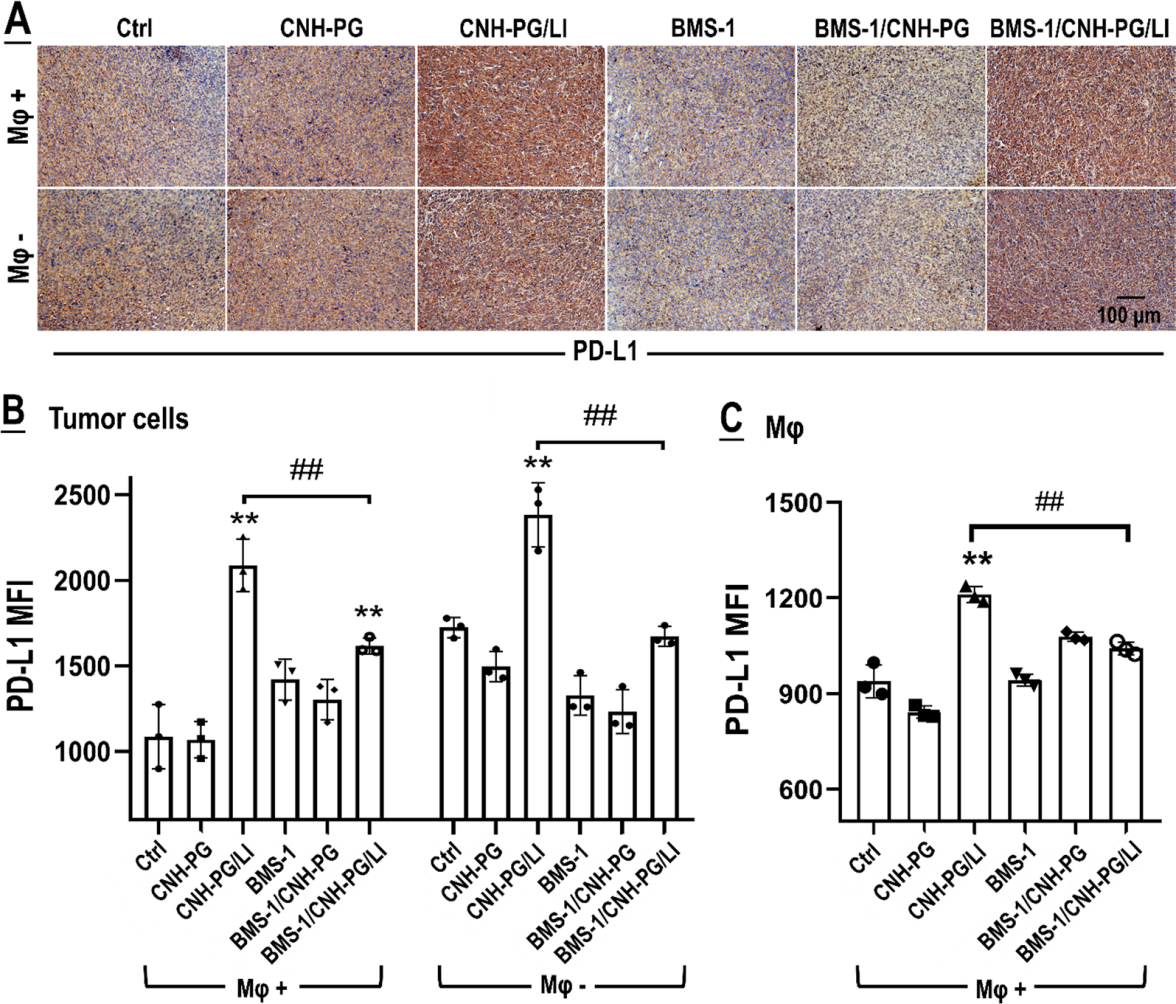
PD-L1 expression in the tumors with or without TAMs depletion. **A** Tumor tissue sections showing IHC staining of PD-L1. Staining of PD-L1 appeared as dark brown spots. **B**, **C** Cell surface PD-L1 expression in the tumor cells and TAMs assayed via immunofluorescent staining and flow cytometry. Values are means ± standard deviation (SD). (n = 3, ## & **p < 0.01). Representative flow cytometry dot plots/histograms are provided in Additional file 1: Fig. S7

**Fig. 10:**
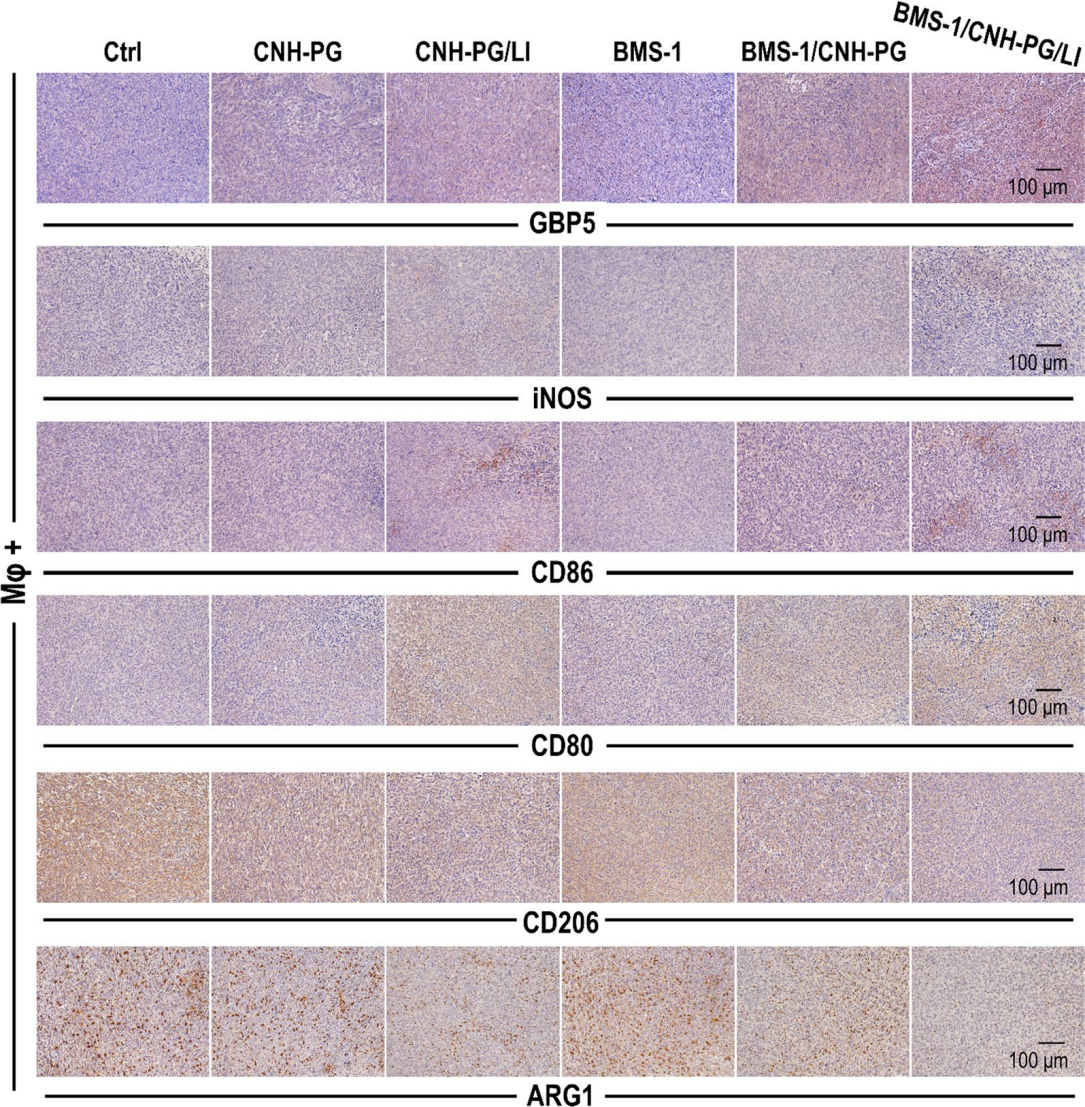
TAMs phenotype in the tumors without TAMs depletion. Tumor tissue sections showing IHC staining of markers of M1-like activation (GBP5, iNOS, CD86, CD80) and markers of M2-like activation (CD206, ARG1). Staining of GBP5, iNOS, CD86 and CD80 appeared as dark brown spots

The impact of macrophage depletion on PD-L1 expression in the tumor tissues is shown in Fig. 9A. CNH-PG-mPT resulted in marked PD-L1 upregulation in the tumor tissue irrespective of macrophage depletion. However, the CNH-PG-mPT-induced PD-L1 upregulation was to an appreciably lesser extent in tumor tissues with depleted macrophages (Mφ−) than in tumor tissues not depleted of macrophages (Mφ +), indicting the TAM to be a major source of PD-L1 expression. Additionally, treatment of BMS-1 palpably alleviated the CNH-PG-mPT-induced PD-L1 upregulation in the tumor tissues irrespective of macrophage depletion. Regardless of TAMs depletion, the 4T1 tumors subjected to CNH-PG-mPT exhibited marked PD-L1 upregulation in the tumor cells, which was alleviated by BMS-1 (Fig. 9A, B). TAMs in the tumors subjected to CNH-PG-mPT also displayed PD-L1 upregulation which was repressed by BMS-1 as well (Fig. 9C). CNH-PG-mPT markedly increased the presence of M1-like macrophages in the 4T1 tumors as indicated by the intensified staining of GBP5, iNOS, CD86, CD80, and alleviated staining of CD206 and ARG1, and these changes were more striking in tumors that received CNH-PG-mPT plus BMS-1 (Fig. 10). The above changes agreed well with in vivo observations presented in Fig. 3B–D and in vitro observations presented in Figs. 4, 5, 6 as well.

## Discussion and conclusions

The first and foremost finding of this work was that targeted CNH-PG-mPT (43–45 ℃) for a short duration of 10 min represents a definite stress to the 4T1 tumors eliciting distinct responses from the tumor cells and the TAMs. The 4T1 tumor cells show a low level of cytotoxicity while the macrophages exhibited a phenotypic shift toward the anti-tumor M1-like polarization instead of cytotoxicity. Intriguingly, PD-L1 upregulation was a common response both in the 4T1 cells and TAMs, and PD-L1 blockade by a non-toxic dose of BMS-1 palpably potentiated the effects of CNH-PG-mPT both on the tumor cells (toxicity) and TAMs (repolarization). These observations firmly substantiate the proposition that PD-L1 plays differentiated intrinsic roles in the tumor cells (pro-survival) and their TAMs (immunosuppressive) [27, 28, 47, 48]. Notably, CNH-PG-mPT also induced PD-1 in the TAMs, which gave the tumor cells an external handle to check the TAMs repolarization via PD-L1/PD-1 binding, blockade of which would further augment the M1-like repolarization of TAMs. Indeed, BMS-1 only displayed a weak synergy with CNH-PG-mPT to elicit TAM repolarization and tumor cell toxicity when the tumor cells and the TAM were in a single culture, and this synergy became more pronounced when the two cell populations were in co-culture. Taken together, the blockade of mPTT‑induced PD‑L1 achieves synergistic anti-TNBC efficacy by taking the intrinsic survival edge off the tumor cells on one hand and taking the brakes off the M1-like TAMs on the other. Interestingly, although BMS-1 both enhanced CNH-PG-mPT’s toxicity to the tumor cells and CNH-PG-mPT-induced TAMs repolarization, the synergetic therapeutic efficacy of BMS-1 and CNH-PG-mPT seemed to largely depend on the presence of TAMs. This finding highlights the significance and potential of TAMs reprogramming as an effective therapeutic strategy against tumor, and tumor-targeted mild photothermia in combination with PD-L1 blockade, as presented in this work, represents a novel and practicable approach thereto. Direct tumor cell toxicity by the repolarized TAMs, as demonstrated in our case, is likely only part of the picture where TAM reprogramming contributes to tumor suppression. TAMs are central coordinators of the activities of various other non-malignant cells for the formation of a microenvironment that promotes tumor cell survival and growth and suppresses the infiltration and activation of other immune components e.g. the dendritic cells and lymphocytes [10–13]. TAM reprogramming may disrupt this tumor-favoring microenvironment and change it into one that permits or favors the anti-tumor immunity mediated by other immune cells [10–13]. It is a limitation of the present work that we have not explored these aspects.

Although the present work successfully obtained tumor-targeted thermia through photothermal heating, other non-invasive and targeted heating methods like microwave and ultrasound are likely to work as well. We also conducted in vitro control experiments wherein warm-air heating was adopted to obtain mild thermia and achieved similar results to those obtained with photothermia (data not shown). For achieving photothermia, other biocompatible photothermal converting agents such as black phosphorus, polydopamine, and gold nanoparticles should also be useful. The CNH used in our work are a new type of nanocarbons that consist of single-walled nanocones of sp^2^-bonded carbon atoms with a diameter of 2–5 nm and a length of 30–50 nm. Unlike carbon nanotubes, CNH have higher purity and improved biocompatibility as their synthesis does not require toxic metal catalysts. Due to their unique molecular structure and morphology, CNH possess strong absorption in the near-infrared (NIR) region, rendering CNH an ideal photothermal agent [49]. The porous morphology of CNH affords a large specific surface area and provides both interior space and exterior surfaces for cargo loading. Moreover, oxidized CNH have many openings on the nanocones with hydroxyl and carboxyl groups on the edges, which not only increase hydrophilicity but also facilitate further modifications such as PG decoration [50]. With these amenable properties, CNH have emerged as a promising biomaterial with application potential in drug delivery [51, 52], bioimaging [53], and photothermal conversion [54–56]. We have previously reported the application of CNH-PG as a theranostic nanodevice for photoacoustic imaging and effective radiochemotherapy of cancer [42]. Direct drug delivery into tumors has emerged as an administration route with some outstanding advantages including high tumor drug availability, low systemic toxicity, and efficient induction of robust anti-tumor responses for immunotherapies. A wide array of tumor immunotherapies are under development adopting local injection as an administration route [49–53, 57–59]. In this work, tumor-targeted photothermia was efficiently obtained through LI of intratumorally injected CNH-PG. In light of the synergy of CNH-PG-mPT and BMS-1 demonstrated in the present work, it is reasonable to expect a higher synergetic anti-tumor efficacy of intratumorally injected CNH-PG that deliver BMS-1 and mediate mPT at the same time. Further study is underway looking into this possibility. Another limitation of the present work is that the mechanism was not explored whereby CNH-PG-mPT for merely 10 min induces conspicuous PD-L1 upregulation both in the tumor cells and TAMs. Further study on this question has been planned. BMS-1, the PD-L1 blocker used in our work, is among a series of structurally related small molecule agents that can induce PD-L1 dimerization and thereby block its interaction with PD-1 [60–62]. Compared with antibody-based PD-L1 blockers, small molecule PD-L1 blocking agents are much less costly and more amenable to incorporation in a nanoparticle.

In conclusion, our work has revealed PD-L1 upregulation to be a key response of TNBC to mild thermal stress, which plays a pro-survival role in the tumor cells while also acting as a brake on the M1-like activation of the TAMs. Blockade of the induced PD‑L1 achieves synergistic anti-TNBC efficacy through both enhancing tumor cell toxicity and potentiating TAMs-mediated anti-tumor response. Our findings reveal a novel way (i.e. mild thermia plus PD-L1 blockade) to modulate the TAMs-tumor cell interaction to instigate a mutiny of the TAMs against their host tumor cells.

### Supplementary Information

**Additional file 1: Table S1.** Normalized data of TAM phenotyping and 4T1 toxicity from Figs. 4, 5 & 6 showing the synergy of BMS-1 with CNH-PG-mPT in single culture and co-culture. **Fig. S1.** Macrophage depletion in the tumor confirmed by A, B: immunofluorescent staining and flow cytometry of F4/80 and CD11b; C: IHC analysis of F4/80 and CD11b. D: Photos of subcutaneous tumor in BALB/c nude mice at 4 h after CNH-PG injection. E: Thermal imaging of subcutaneous 4T1 tumors injected with about 20 μL of CNH-PG solution (5 mg/mL) that was subjected to 808 nm LI (0.78 W/cm^2^, a light spot 1 cm in diameter) for 10 min at 4 h post injection. G: Photos of subcutaneous tumor in BALB/c mice at 4 h after CNH-PG injection. **Fig. S2.** Quantitative analysis of immunohistochemistry and immunofluorescence in Figure 3. A: The proportion of positive staining area (% area) of PCNA, Ki67 and Cleaved-caspase3 in Figure 3A. This result was obtained using the IHC Toolbox plug-in of ImageJ software to measure six randomly selected fields of view. B-D: The mean fluorescence intensity (MFI) of positive staining for PD-L1, CD86 and CD206 in Figure 3B-D. The results were obtained using the ImageJ software to measure fluorescence intensity in the positive regions of six randomly selected visual fields. **Fig. S3.** Grayscale analysis of immunoblot in Figure 4C and Figure 5B. The quantitative analysis was measured by ImageJ software. The vertical axis shows the ratio of the target protein to the internal reference protein GAPDH (n = 3, # & *p < 0.05, ## & **p < 0.01). **Fig. S4.** Representative flow cytometry FSC/SSC, dot plots/histograms and gating strategy for data presented in Fig. 4D, F, G, and I. **Fig. S5.** Representative flow cytometry FSC/SSC, dot plots/histograms and gating strategy for data presented in Fig. 5C-F, H and I. **Fig. S6.** Representative flow cytometry FSC/SSC, dot plots/histograms and gating strategy for data presented in Fig. 6A-I. **Fig. S7.** Representative flow cytometry FSC/SSC, dot plots/histograms and gating strategy for data presented in Fig. 9B and C. **Fig. S8.** Original data of western blots presented in Fig. 4C. **Fig. S9.** Original data of western blots presented in Fig. 4H. **Fig. S10.** Original data of western blots presented in Fig. 5B. **Fig. S11.** Original data of western blots presented in Fig. 5G.

## Data Availability

All data generated or analyzed during this study are included in this published article and its additional information files.
