# Supplementary material for: PD-L1 blockade TAM-dependently potentiates mild photothermal therapy against triple-negative breast cancer
Source: J Nanobiotechnology. 2023 Dec 11;21:476. doi: 10.1186/s12951-023-02240-3 (PMC10712197; doi:10.1186/s12951-023-02240-3)
Supplement: Supplementary file 1 — Additional file 1: Table S1. Normalized data of TAM phenotyping and 4T1 toxicity from Figs. 4, 5 & 6 showing the synergy of BMS-1 with CNH-PG-mPT in single culture and co-culture. Fig. S1. Macrophage depletion in the tumor confirmed by A, B: immunofluorescent staining and flow cytometry of F4/80 and CD11b; C: IHC analysis of F4/80 and CD11b. D: Photos of subcutaneous tumor in BALB/c nude mice at 4 h after CNH-PG injection. E: Thermal imaging of subcutaneous 4T1 tumors injected with about 20 μL of CNH-PG solution (5 mg/mL) that was subjected to 808 nm LI (0.78 W/cm2, a light spot 1 cm in diameter) for 10 min at 4 h post injection. G: Photos of subcutaneous tumor in BALB/c mice at 4 h after CNH-PG injection. Fig. S2. Quantitative analysis of immunohistochemistry and immunofluorescence in Figure 3. A: The proportion of positive staining area (% area) of PCNA, Ki67 and Cleaved-caspase3 in Figure 3A. This result was obtained using the IHC Toolbox plug-in of ImageJ software to measure six randomly selected fields of view. B-D: The mean fluorescence intensity (MFI) of positive staining for PD-L1, CD86 and CD206 in Figure 3B-D. The results were obtained using the ImageJ software to measure fluorescence intensity in the positive regions of six randomly selected visual fields. Fig. S3. Grayscale analysis of immunoblot in Figure 4C and Figure 5B. The quantitative analysis was measured by ImageJ software. The vertical axis shows the ratio of the target protein to the internal reference protein GAPDH (n = 3, # & *p < 0.05, ## & **p < 0.01). Fig. S4. Representative flow cytometry FSC/SSC, dot plots/histograms and gating strategy for data presented in Fig. 4D, F, G, and I. Fig. S5. Representative flow cytometry FSC/SSC, dot plots/histograms and gating strategy for data presented in Fig. 5C-F, H and I. Fig. S6. Representative flow cytometry FSC/SSC, dot plots/histograms and gating strategy for data presented in Fig. 6A-I. Fig. S7. Representative flow cytometry FSC/SSC, dot plots/hi [file 12951_2023_2240_MOESM1_ESM.docx]

**Table S1. Normalized data of TAM phenotyping and 4T1 toxicity from Fig 4, 5 & 6 showing the synergy of BMS-1 with CNH-PG-mPT in single culture and co-culture.**

|  | **Single culture**  **(Fig. 4 & 5)** | **Co-culture**  **(Fig. 6)** |
| --- | --- | --- |
| **TAM phenotyping** |  |  |
| CD80 MFI | 100.7±0.5 | 107.9±0.6** |
| CD86 MFI | 98.0±1.2 | 114.1±1.9** |
| CD206 MFI | 117.9±1.7 | 94.4±1.9** |
| Percent of positive cell (%) | 103.6±2.2 | 106.2±0.5** |
| Beads MFI of positive cell | 139.9±0.1 | 152.8±1.4* |
|  |  |  |
| **4T1 toxicity** |  |  |
| CFSE MFI | 108.0±0.8 | 114.7±0.3** |
| Cell of death (%) | 107.6±4.2 | 126.9±5.3* |

**Synergy of BMS-1 and CNH-PG-mPT**S is evaluated as follows:

$Synergy or Addition=\frac{Value for CNH-PG/LI (1.5 W/cm2)/BMS-1}{Value for CNH-PG/LI (1.5 W/cm2) \boldsymbol{+} Value for BMS-1\boldsymbol{-} Value for Control}$ *× 100%*

There is a synergy when the outcome is above 100%, otherwise it is addition.

(n = 3, **p* < 0.05, ***p* < 0.01).


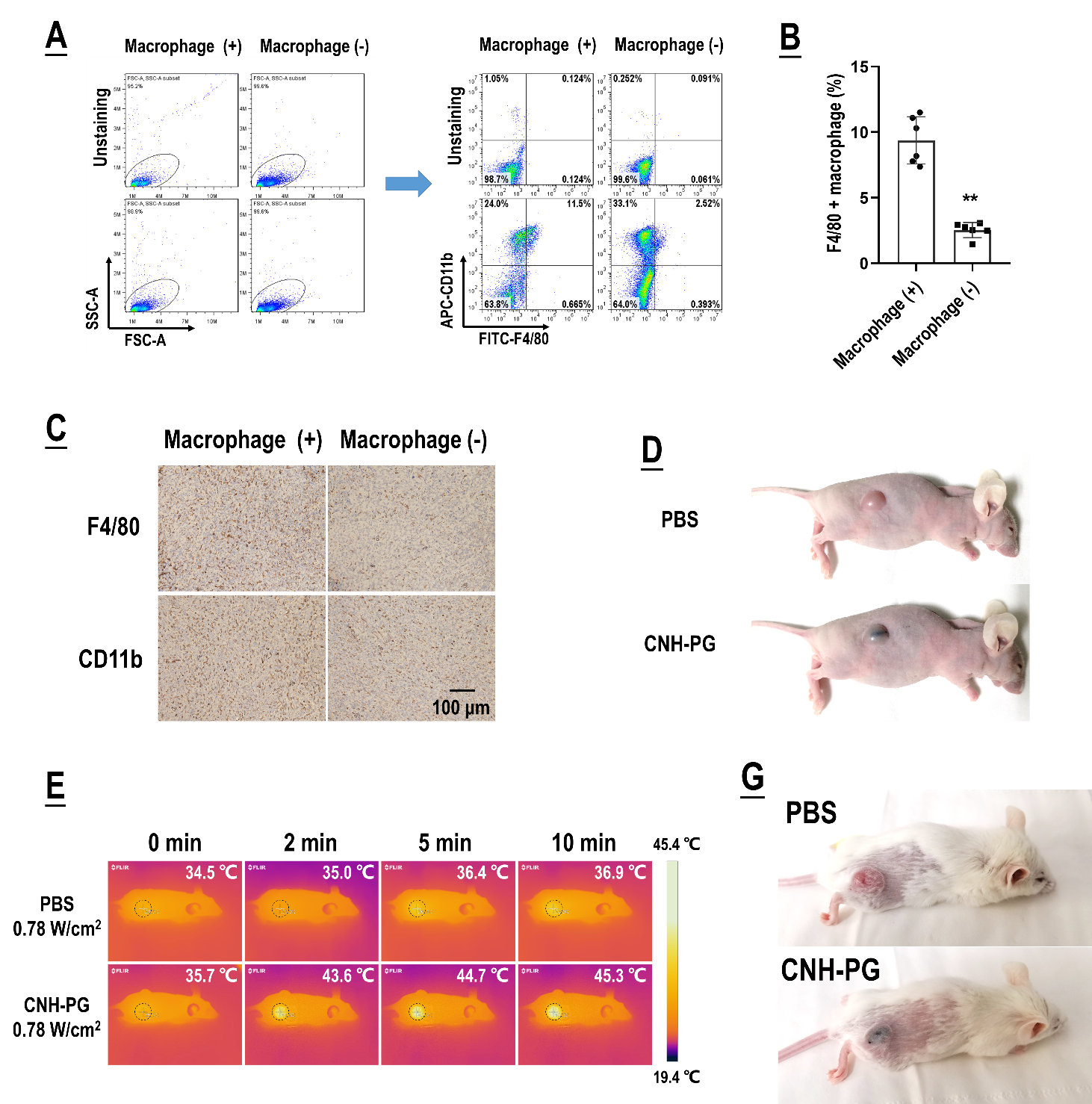


**Fig. S1.** Macrophage depletion in the tumor confirmed by **A, B:** immunofluorescent staining and flow cytometry of F4/80 and CD11b; **C:** IHC analysis of F4/80 and CD11b. **D**: Photos of subcutaneous tumor in BALB/c nude mice at 4 h after CNH-PG injection. **E**: Thermal imaging of subcutaneous 4T1 tumors injected with about 20 μL of CNH-PG solution (5 mg/mL) that was subjected to 808 nm LI (0.78 W/cm^2^, a light spot 1 cm in diameter) for 10 min at 4 h post injection. **G**: Photos of subcutaneous tumor in BALB/c mice at 4 h after CNH-PG injection.


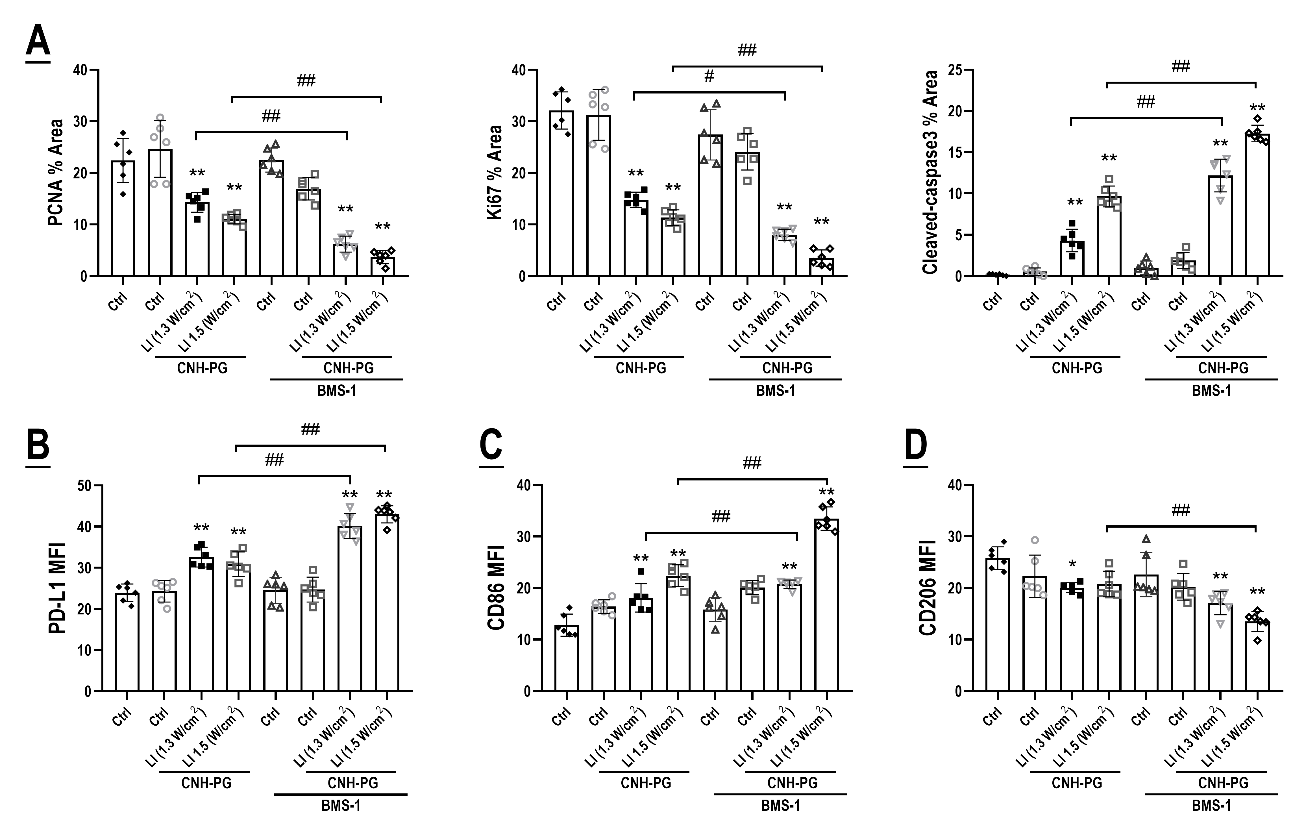


**Fig. S2. Quantitative analysis of immunohistochemistry and immunofluorescence in Figure 3.** **A**: The proportion of positive staining area (% area) of PCNA, Ki67 and Cleaved-caspase3 in Figure 3 A. This result was obtained using the IHC Toolbox plug-in of ImageJ software to measure six randomly selected fields of view. **B-D**: The mean fluorescence intensity (MFI) of positive staining for PD-L1, CD86 and CD206 in Figure 3 B-D. The results were obtained using the ImageJ software to measure fluorescence intensity in the positive regions of six randomly selected visual fields.


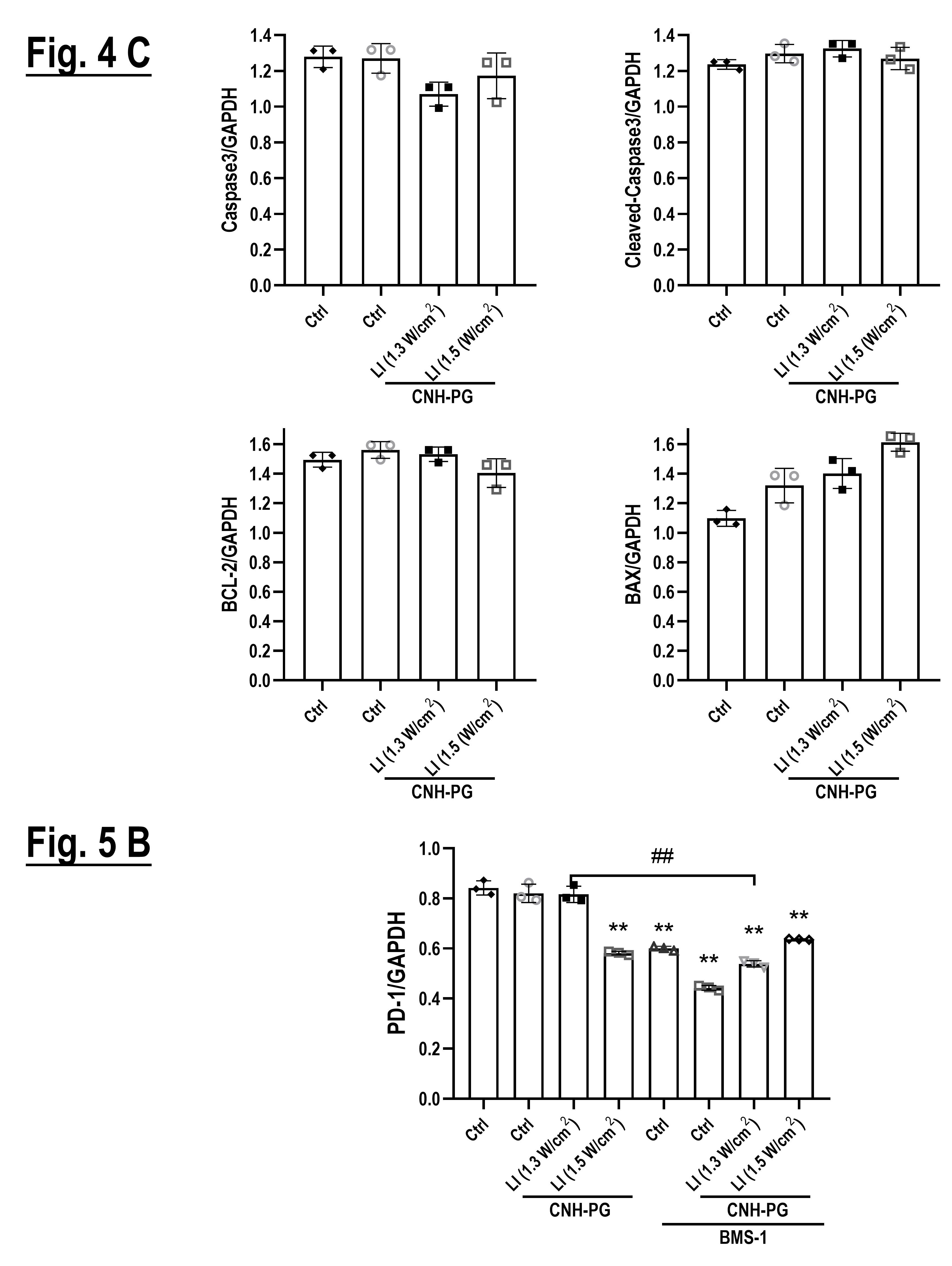


**Fig. S3. Grayscale analysis of immunoblot in Figure 4 C and Figure 5 B.** The quantitative analysis was measured by ImageJ software. The vertical axis shows the ratio of the target protein to the internal reference protein GAPDH (n = 3, # & **p* < 0.05, ## & ***p* < 0.01).


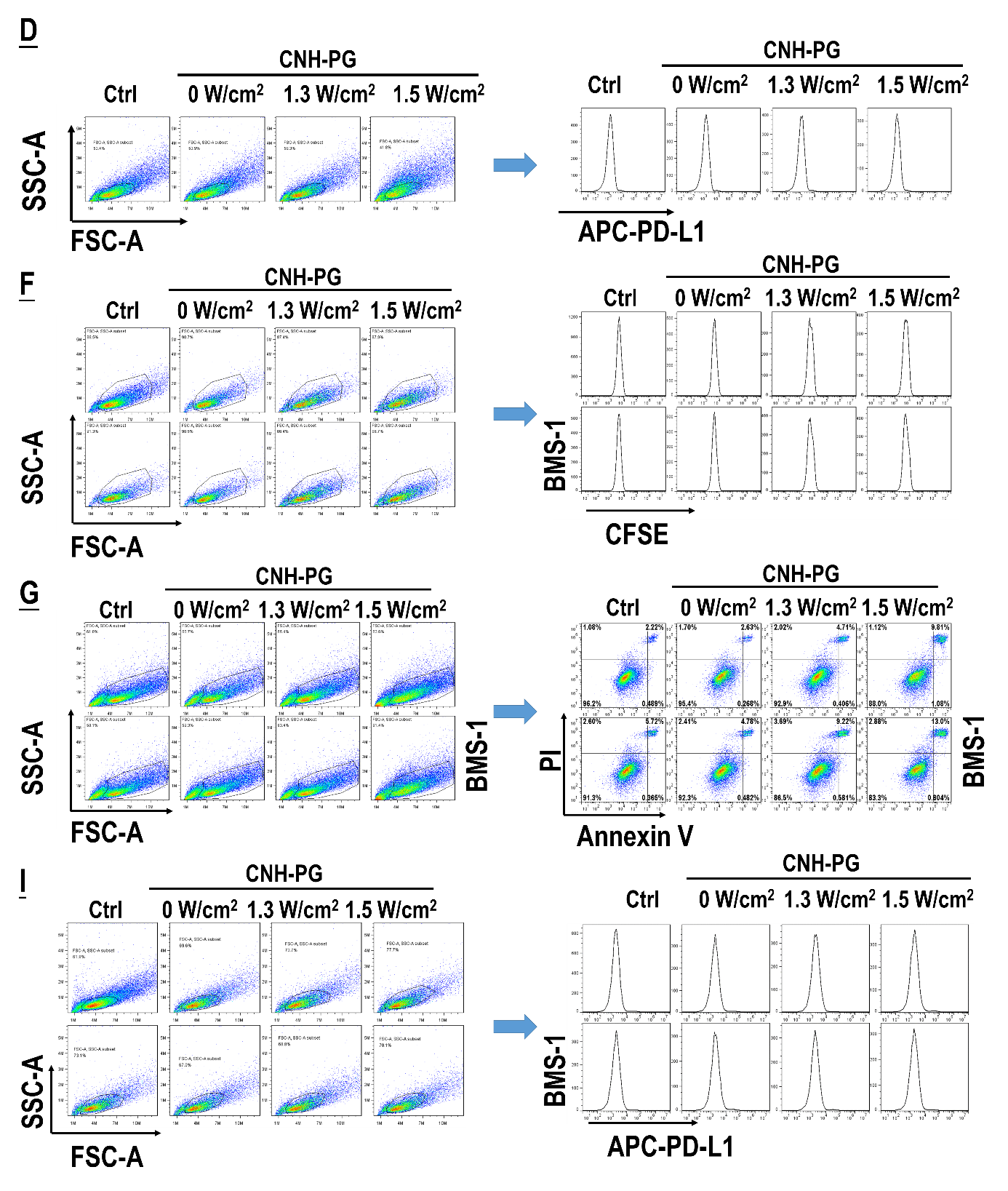


**Fig. S4.** **Representative flow cytometry FSC/SSC, dot plots/histograms and gating strategy for data presented in Fig. 4 D, F, G, and I.**

**
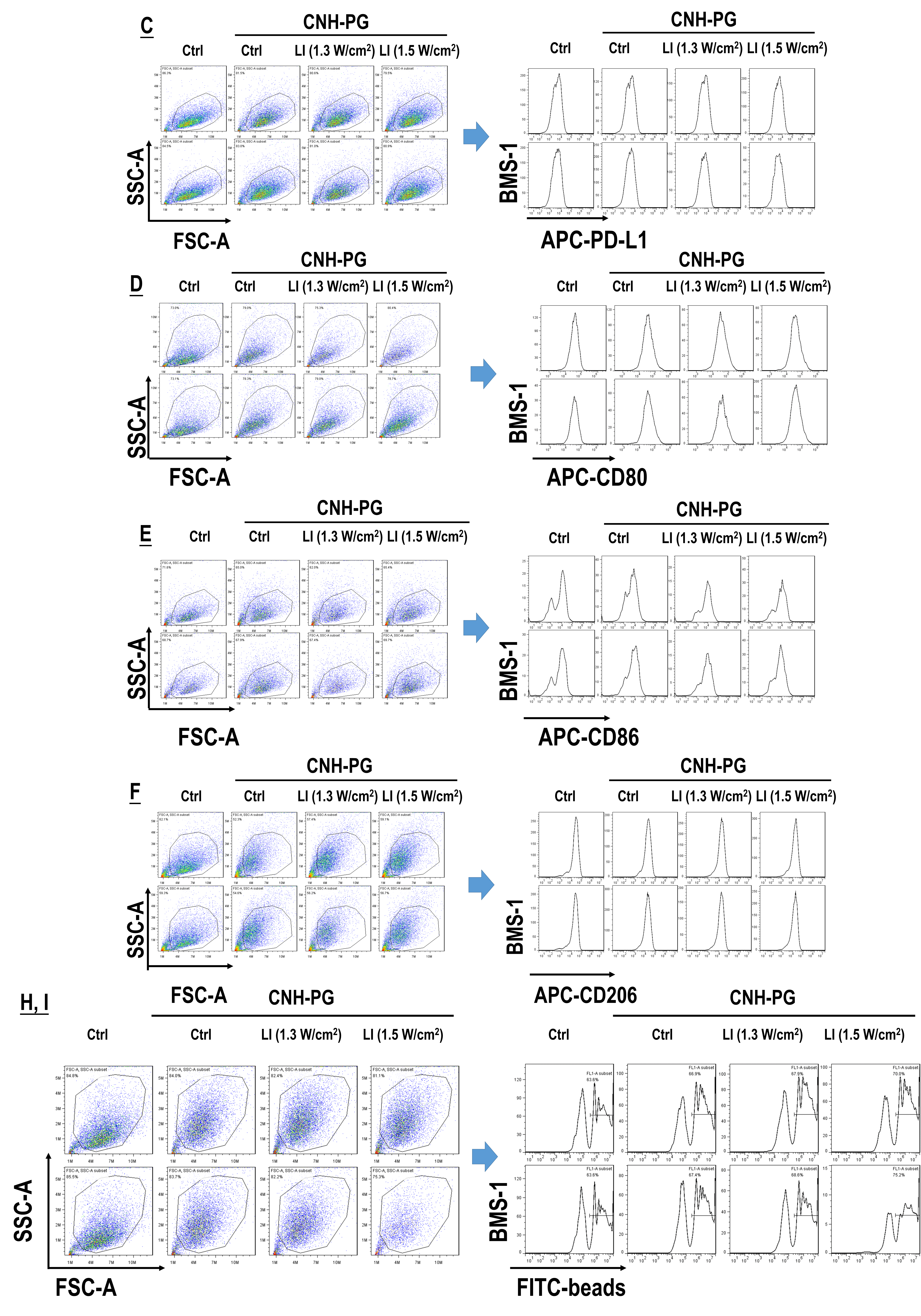
**

**Fig. S5. Representative flow cytometry FSC/SSC, dot plots/histograms and gating strategy for data presented in Fig. 5 C-F, H and I.**

**Fig. S6.** **Representative flow cytometry FSC/SSC, dot plots/histograms and gating strategy for data presented in Fig. 6 A-I.**


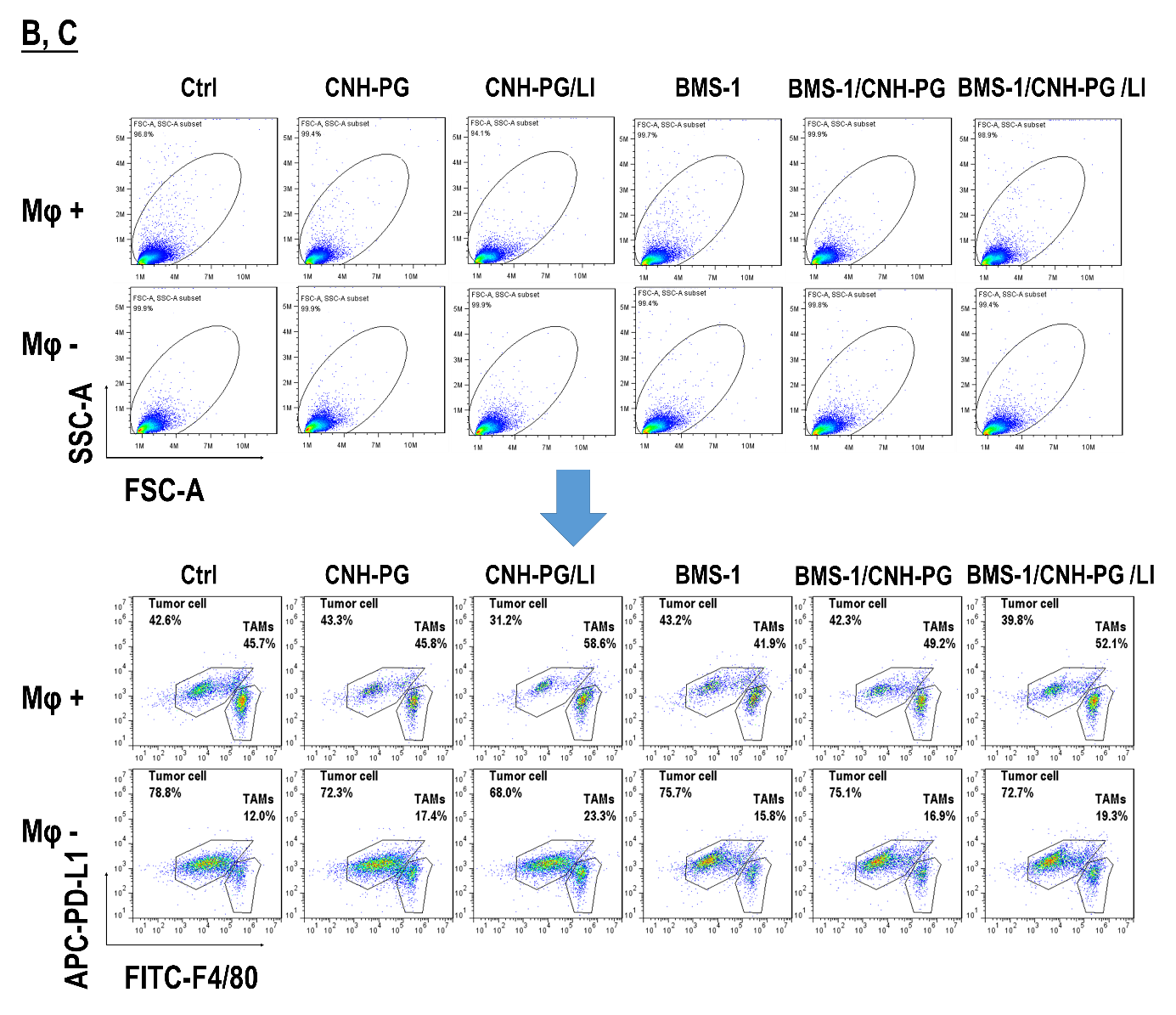


**Fig. S7. Representative flow cytometry FSC/SSC, dot plots/histograms and gating strategy for data presented in Fig. 9 B and C.**

**Fig. S8. Original data of western blots presented in Fig. 4 C.**

**Fig. S9. Original data of western blots presented in Fig. 4 H.**

**Fig. S10. Original data of western blots presented in Fig. 5 B.**

**Fig. S11. Original data of western blots presented in Fig. 5 G.**
